# Co-designing PROFESS: a community health worker support framework to strengthen engagement in TB and HIV care in Madagascar

**DOI:** 10.3389/frhs.2026.1910840

**Published:** 2026-09-18

**Authors:** Onja Gabrielle Ravololohanitra, Haingotiana Razafimanantsoa, Larissa Hecht, Michel Tiaray Harison, Norosoa Assunta Felananirina Rakotoherimora, Harizaka Emmanuel Andriamasy, Nadine Muller

**Affiliations:** 1 Doctors for Madagascar, Research and M&E, Antananarivo, Madagascar; 2 Global Digital Last Mile Health Research Lab at Charité Center for Global Health, Charité - Universitätsmedizin Berlin, Berlin, Germany; 3 Speciality Network: Infectious Diseases and Respiratory Medicine, Charité - Universitätsmedizin Berlin, Berlin, Germany; 4 Ärzte für Madagaskar e.V., Leipzig, Germany; 5 Programme National de Lutte Contre la Tuberculose, Antananarivo, Madagascar; 6 Programme National de Lutte Contre les IST et le Sida, Antananarivo, Madagascar

**Keywords:** co-design, community health workers, design thinking, health system fragmentation, implementation, service integration, TB/HIV

## Abstract

Community health workers (CHWs) are central to primary health care in low- and middle-income countries, yet their engagement is often uneven across health domains. In Madagascar, approximately 50,720 CHWs serving on a voluntary, unsalaried basis link communities to the formal health system, but only a minority is actively engaged in tuberculosis (TB) or HIV service delivery despite substantial disease burden. This community case study describes a two-stage initiative by a consortium of government disease programmes, an NGO, and an academic partner to strengthen CHW engagement in TB and HIV care in Madagascar. Stage 1 combined a cross-sectional survey of CHWs across four regions with a nested qualitative component identifying system-level constraints. Stage 2 convened national, sub-national, and community-based stakeholders across sectors in a three-day design-thinking workshop, producing PROFESS, a seven-lever framework operationalised as a guide for strengthening CHW support including concrete measures related to Protection (financial), Recognition, Coordination, Formation (training), Equipment, Supervision, and Security of CHWs. PROFESS has been validated by the Malagasy Ministry of Public Health and published as an operational guide. This case highlights two key lessons for fragmented, evidence-scarce settings: workforce-support frameworks anchored in foundational system functions are transferable across disease domains, and co-design produces institutionally adoptable outputs only when paired with operational elaboration. Remaining challenges include the definition of minimum support packages and the sequencing of implementation across levers. A pilot implementation of PROFESS is currently underway, with first results expected in 2027.

## 1 Introduction

Community health workers (CHWs), a heterogeneous set of cadres ranging from community-selected volunteers working without employment contract to salaried formally integrated national health workforces, are central to primary health care, particularly in low- and middle-income settings where workforce shortages and geographic barriers limit access to facility-based services (1). National policies in many such settings task CHWs with responsibilities across multiple health domains, including high-burden infectious diseases such as tuberculosis (TB) and HIV (2). In practice, however, CHW engagement across these domains is uneven. Limited foundational support and the socioeconomic vulnerability inherent in volunteer arrangements often constrain CHWs to programme- or domain-specific engagement rather than the full range of expected responsibilities (3, 4). Engagement in TB and HIV care, in particular, often lags behind engagement in maternal and child health, immunisation, or malaria (5, 6). TB and HIV have been described to be particularly challenging domains for community-level engagement as both involve persistent stigma, both disproportionately affect populations of low socioeconomic status that are typically harder to reach through community-based services, and TB carries higher occupational infectious risk for CHWs, amplified where equipment, training, and supervision are inconsistent (7).

In many settings, efforts to strengthen CHWs frequently take the form of time-limited, isolated initiatives, such as disease-specific task shifting, refresher training tied to a single condition, or stand-alone incentive schemes attached to particular health domains (8). As a result, strengthening CHW engagement in one domain often fails to translate into engagement in others, and may even crowd out engagement elsewhere in the CHW's portfolio (9, 10). Strengthening CHW engagement in under-served disease areas such as TB and HIV therefore requires attention to the underlying workforce-support architecture, rather than additional disease-specific layers. Practical, context-adapted frameworks that align actors and define the minimum support package CHWs require are particularly needed in settings where workforce evidence is scarce and partner-driven implementation produces fragmented support.

Closing this gap requires approaches that account for health-system complexity and align actors operating under different mandates (11). Participatory and co-design methods, including design thinking, are increasingly used in health systems research (12). Compared with consultative or deliberative formats, design thinking is human-centred, iterative, and prototype-oriented; it progresses from a structured understanding of user needs through problem definition and ideation to concrete, testable solutions. Although design thinking has been applied to clinical pathways and digital health tools (13), its use to develop system-level CHW support remains limited.

This community case study describes how a cross-sector consortium in Madagascar combined primary evidence generation with multi-stakeholder co-design to develop a multi-lever support architecture named PROFESS. The paper aims to (1) describe the approach used to move from formative evidence to PROFESS, a seven-lever support framework; (2) present the framework's operational guidance; and (3) identify lessons for strengthening CHW support in settings where workforce evidence is scarce and engagement in specific disease areas remains low.

## 2 Context: community health workers in Madagascar

Madagascar is a low-income country of approximately 32 million people (14). With fewer than 2 physicians per 10,000 population, its health-workforce density is among the lowest globally (15). About 68% of the population live in rural areas and access to health care is substantially limited by high geographical distance and limited household income (16, 17). Approximately 50,720 voluntary CHWs form the interface between communities and the formal health system, corresponding to roughly one CHW per 630 inhabitants across 17,485 *fokontany* (villages), the smallest administrative unit (18). They are locally known as *agents communautaires* (“AC”), are selected by the communities they serve, act on a voluntary basis by national directive, and hold no employment contract with the Ministry of Public Health. They are primarily attached to basic health centres (Centres de santé de base; CSB) and engage in a broad portfolio of activities including health promotion, disease prevention, referral, and follow-up across maternal and child health, TB, HIV, malaria, nutrition, water and sanitation, and other domains. National policies, such as the *Politique Nationale de Santé Communautaire* (19) and the operational Guide Paquet d'Activités Communautaires (Guide PAC) (20) define CHW roles and responsibilities across health domains. The Guide PAC organises these responsibilities into three tiers: a basic package including communication and sensitisation expected of all CHWs, an essential package of high-impact interventions expected of all CHWs, and a specific package of activities requiring additional competencies (20). Malaria is anchored in the essential package, through *PCIME communautaire*, Madagascar's operationalisation of integrated community case management, and through routine prevention in pregnancy; TB is placed in the specific package and is therefore conditional on additional training; and HIV appears as prevention of mother-to-child transmission within antenatal care in the essential package, with no comprehensive community-level HIV framework beyond this. While these policies are clear on what CHWs are expected to do, they provide less precision and details on how their work shall be supported and enabled in practice. Supervision, compensation, training, and logistical inputs are therefore largely defined at programme and partner level, and remain inconsistently operationalised in practice. This pattern partly reflects how CHW programming is financed in Madagascar. Direct government funding for CHW activities is limited; CHW support is therefore heavily dependent on external funding, primarily from non-governmental organisations (NGOs). Government programmes, NGOs, and disease-specific initiatives consequently operate within their respective priority domains, with separate planning, funding, and reporting structures, producing fragmented implementation across regional, district, and commune levels, down to individual CHWs. The consequences of this fragmentation are most visible in domains that receive limited priority from either government or external funders, such as TB and HIV. Both domains carry a substantial and, in the case of HIV, growing burden: the estimated TB incidence has remained stable at 233 per 100,000 since 2013 (21); and an estimated 76,000 people in Madagascar were living with HIV in 2023 with growing evidence of a generalised HIV epidemic (22, 23), prompting repeated national multi-sectoral calls to action (24, 25).

At the time this initiative was launched, the extent of CHW involvement in TB and HIV service delivery was not empirically established. Both national programmes considered CHW engagement to be low, based on programme monitoring and key informant knowledge. National surveillance indicated that CHW-initiated referrals accounted for no more than 12% of TB case notifications, although no representative data existed to confirm, quantify, or explain this. Peer-reviewed evidence on CHW needs and programming conditions in Madagascar was limited to one cross-sectional survey of CHWs across 5 regions in 2015, documenting CHWs' precarious livelihoods, limited benefits received and life-work balance, and underscoring tensions between expanding CHW responsibilities and the constrained context in which CHWs are expected to operate (26).

## 3 Key elements: development of PROFESS

### 3.1 Initiative and consortium

The initiative was developed through a cross-sector partnership between the Programme National de Lutte contre la Tuberculose and the Programme National contre les IST et le Sida (National TB and National sexually transmissible infections & HIV/AIDS Programmes, Malagasy Ministry of Public Health), the NGO Doctors for Madagascar, and the academic partner Charité - Universitätsmedizin Berlin, building on a long-standing collaboration. The initiative linked empirical evidence with participatory design through a two-stage process: (i) formative assessment of the CHW system, and (ii) co-design of a support framework. TB and HIV were selected as priority focus areas, reflecting both their epidemiological burden in Madagascar and a particularly low CHW engagement in disease response activities estimated by both national programmes. Primary evidence generation was therefore an explicit first stage activity of the initiative towards designing adequate support.

### 3.2 Stage 1: formative assessment of CHW roles and system constraints

Formative research was conducted between March and October 2024 to characterise CHW roles, working conditions, and engagement across health domains, with the quantitative component conducted from March to June and the qualitative component in September to October due to logistical considerations and team availability. The formative component of the case study comprised evidence-generation activities conducted under the same initiative and ethical framework (CEER Madagascar 003/2023; Charité - Universitätsmedizin Berlin EA2/083/23). A cross-sectional survey of 408 CHWs (81.8% response rate) was conducted across four regions (Analamanga, Atsimo-Andrefana, Androy, Anosy) using two-stage cluster sampling and an interviewer-administered structured questionnaire covering demographic and professional characteristics, work environment, tasks, training, supervision, and economic aspects (6). The survey questionnaire can be found in Supplementary Material 1. A nested qualitative component was conducted in two contrasting regions (Analamanga, Atsimo-Andrefana). Five focus group discussions (FGDs) were held with 26 CHWs (three in Analamanga, two in Atsimo-Andrefana). Participants were selected through two-stage purposive sampling: CSBs were selected on the basis of TB activity during the preceding 12 months, catchment profile (urban, peri-urban, rural), and logistical accessibility; CHWs at these facilities were eligible if aged 18 years or older, currently active in their role, and engaged in the TB response within the previous 12 months. Group composition was intentionally balanced for gender, tenure, role, and catchment profile. Discussions followed a semi-structured topic guide (Supplementary Material 2), were facilitated in Malagasy and its dialects by trained moderators and note-takers with no prior relationship to participants. They were audio-recorded, transcribed, and translated into French. Transcripts were analysed by reflexive thematic analysis. The full methodology and findings are reported separately (27). In complement, 24 targeted interviews were conducted with 7 facility-based providers, 4 public health authorities, 4 community leaders, 3 field supervisors, and 6 implementing partners, using a semi-structured guide (Supplementary Material 3) covering CHW support arrangements, coordination between actors, and perceived constraints in TB and HIV service delivery.

The formative work confirmed some of the programmes' assumptions, quantified their magnitude, and identified determinants of engagement. Findings from the survey, FGDs, and targeted interviews were brought together to identify cross-cutting, system-level constraints along with the associated needs and recommendations for strengthening CHW engagement. These findings were not treated as predetermined elements of the intervention; rather, they served as the empirical inputs to the co-design phase. Four system-level constraints were especially prominent and shaped the later co-design discussions:

- **Uneven service engagement**: Engagement was measured as self-reported involvement in defined community-level tasks within the preceding two years. Engagement in nationally prioritised activities such as immunisation, maternal health, and malaria was near-universal (99%–100%), whereas only 24% of CHWs reported engagement in TB and 22% in HIV service delivery. Within TB and HIV, engagement was mostly partial: nearly all CHWs active in these domains reported sensitisation activities, whereas referral and treatment follow-up remained uncommon, with fewer than 10% of all CHWs reporting referral for HIV testing. Variation was explained principally by modifiable system-level factors, including possession of written work guidance and receipt of disease-specific training, rather than by CHW characteristics. Reliance on NGO-led support was not associated with increased engagement (6).
- **Socioeconomic vulnerability:** Most CHWs operate under conditions of economic precarity, with 77.7% (317/408) reporting a monthly household income below 100,000 MGA (≈ $22.1) and 92.4% depending on an informal primary occupation as their main livelihood. Overall, CHWs dedicated a median of 6 h per week (IQR 3–11) to their role and 22.8% of CHWs reported receiving no monetary incentive linked to their role in the month preceding the survey (6).
- **Limited supervision**: Supervision was either absent or predominantly administrative in nature, focused on reporting rather than coaching, problem-solving, or technical guidance and support (27).
- **Training and equipment deficits**: Access to standardised training, essential equipment, and transport was inconsistent across regions, with multi-year gaps between sessions reported in some rural areas (27). Essential equipment, personal protective equipment, and means of transport were similarly unevenly provided; in their absence, CHWs frequently improvised, covered shortfalls from personal resources, or missed visits altogether (6, 27).

Alongside these constraints, CHWs and other stakeholders identified a set of recommendations concerning the conditions needed to perform their roles effectively, including more predictable financial support, reliable access to equipment and transport, regularised training, more supportive supervision, and greater recognition and integration within health teams. These constraints and recommendations formed the starting material for the Stage 2 workshop.

### 3.3 Stage 2: participatory co-design of a CHW support framework

To translate Stage 1 findings into operational priorities, a three-day in-person workshop was held near the capital, Antananarivo, in March 2025 with 37 stakeholders spanning system levels and actor types. Participants included CHWs and community supervisors; facility-based health workers, including heads of TB diagnostic and treatment centres (Centres de Diagnostic et de Traitement; CDT) from two contrasting regions; national and regional representatives of the National TB Programme and the National STI & HIV/AIDS Programme; senior Ministry of Public Health staff from the community health and primary care directorates and from regional health authorities; and representatives of multiple non-governmental and faith-based organisations active in community-based TB and HIV support. Recruitment criteria varied by actor type: Programme representatives were invited based on their mandate and decision-making authority over CHW policy; facility-based providers were selected in consultation with regional health authorities; non-governmental and faith-based actors were included for their active roles in CHW support in the study regions; and CHWs were purposively sampled from the Stage 1 roster to reflect diversity in region, sex, and activity profile.

The workshop adapted the standard five-phase design thinking model (empathise, define, ideate, prototype, test) to a three-phase structure suited to a policy co-design context: (1) *empathise and define*, in which Stage 1 findings were presented and CHW personas built from formative data, anchoring discussion in both lived experience and scientific evidence; (2) *ideate*, in which intentionally mixed stakeholder groups generated candidate solutions in response to structured prompts; and (3) *elaborate*, in which prioritised solutions were discussed and specified into concrete, operational actions. The agenda aimed to reach the end of ideation by mid-workshop, freeing the second half for elaboration and discussion of feasible actions. Sessions were facilitated in Malagasy by a trained team. Recognising hierarchical dynamics between the multi-level participants, one facilitator per group was briefed to actively encourage community-level voices and elicit lived experience during group work and plenaries. Additional features sought to support inclusive, evidence-anchored discussion, including intentionally mixed stakeholder group composition, prominent display of Stage 1 key findings and quotes on posters and handouts, short “info-drop” segments presenting good practices and success stories from comparable country settings, and a presentation of an exemplary theory of change-model to encourage out-of-the box thinking.

Prioritisation was achieved through iterative dot voting: participants received colour-coded stickers with preset weights (red = 5, green = 3, yellow = 2) and applied them to proposed levers and actions, with cumulative scores enabling rapid convergence on priorities and dynamic steering of subsequent sessions (Figure 1). Through this process, participants developed, grouped, and prioritised candidate responses, which were then specified into concrete actions clustered under seven operational domains. The seven domains emerged from the co-design process itself, combining the Stage 1 evidence and recommendations with participant's experiential knowledge, priorities, and feasibility judgements, rather than being predefined by the research team.

**Figure 1:**
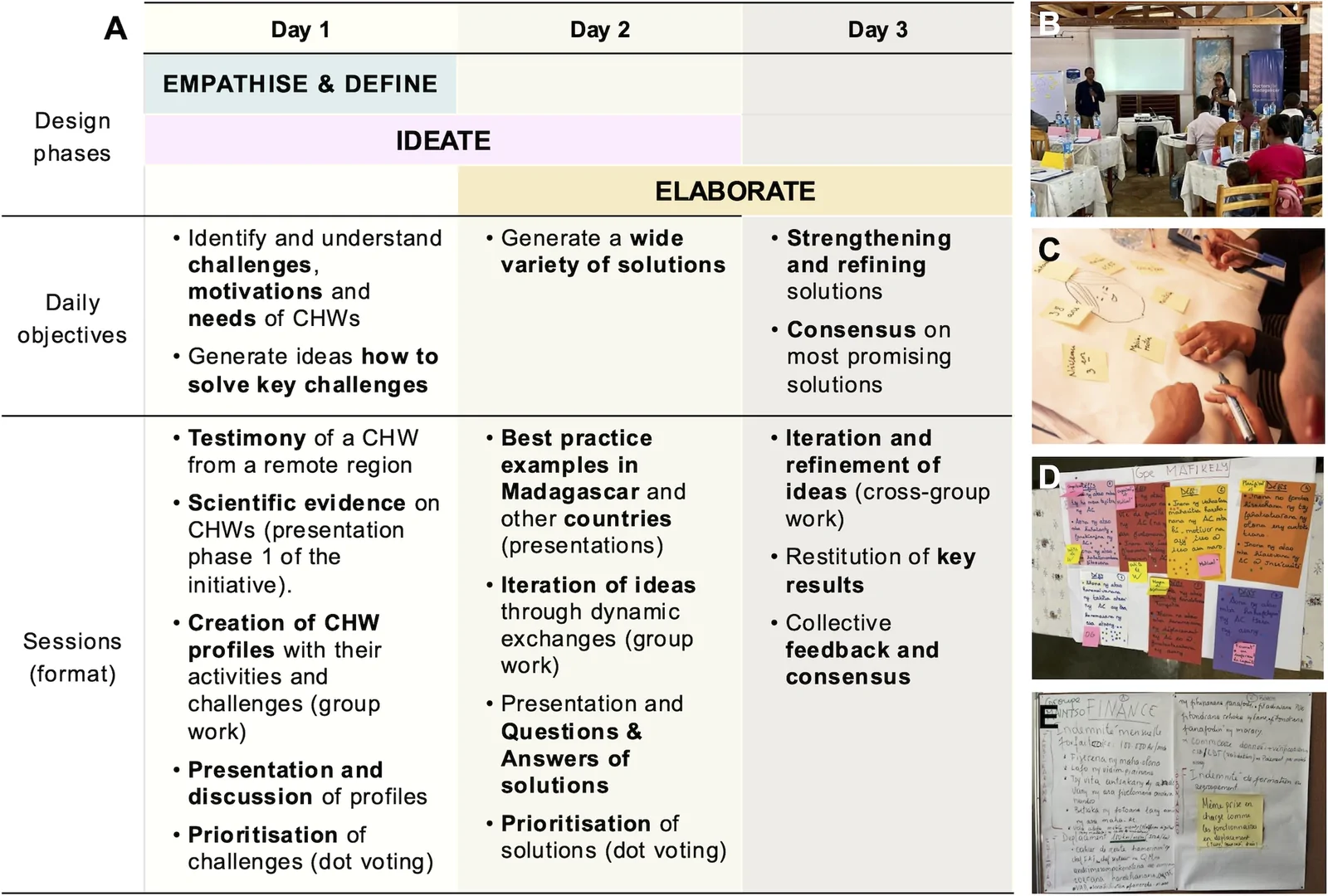
Agenda and impressions from a design-thinking workshop to strengthen community health workers (CHWs), Madagascar, 2025. **(A)** Workshop programme; **(B)** session facilitation; **(C)** creation of CHW personas in group work; **(D)** prioritisation using dot-voting; **(E)** solution draft. CHW, community health worker.

### 3.4 PROFESS: a framework of seven operational levers

The PROFESS acronym and framework were developed by the research team after the workshop as a unifying mnemonic to organise the workshop outputs in a policy-relevant format and were validated by the Ministry of Public Health in March 2026 (28). The original French version is provided in Supplementary Material 4, and Supplementary Material 5 provides an English translation. The resulting framework comprises seven operational levers, each linked to concrete actions: Protection (financial), Recognition, cOordination, Formation (training), Equipment, Supervision, and Security (Figure 2).

**Figure 2:**
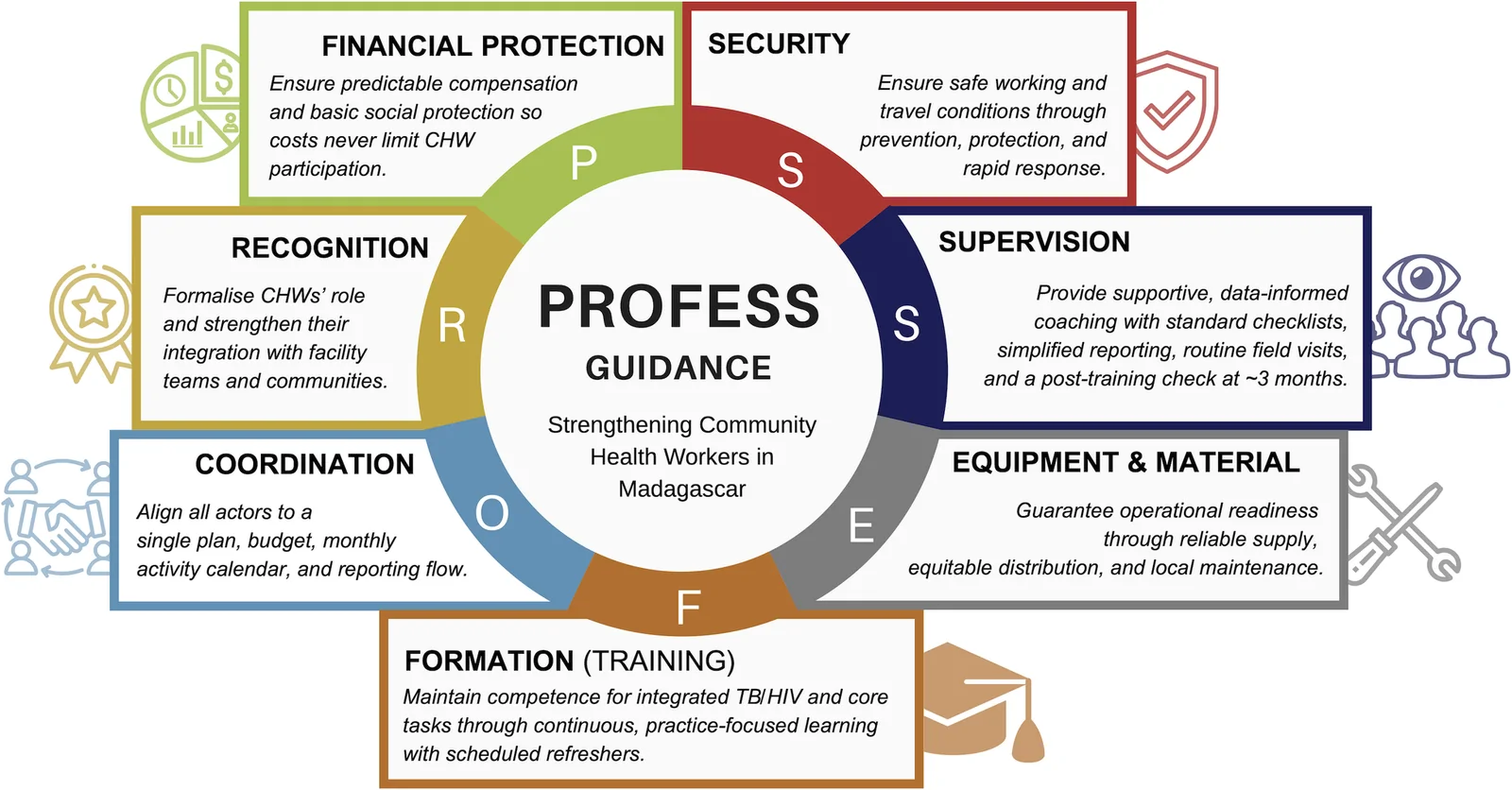
The PROFESS framework: seven operational levers for strengthening community health workers in TB and HIV care in Madagascar, 2025. Detailed operational actions under each lever are provided in Table 1.

**Table 1:** PROFESS levers, goals and recommended actions to strengthen community health worker (CHW) engagement in Madagascar, 2025.

Lever	Goal	Recommended actions
Protection (financial)	Predictable, fair compensation and basic social protection.	• Establish a transparent compensation scale linked to clearly defined expected activities (such as fixed monthly allocation; transport reimbursement if not included; performance bonus only when not covered by the fixed allocation), accompanied by simple factsheets and MoH validation. • Regularise payment processes: agree on the payment channel with CHWs; implement simple digital/registry tracking; publish a monthly payment calendar. • Offer health coverage for CHWs and their dependants (e.g., *mutuelle*, subsidy, equity fund). • Support income generation through CHW associations/cooperatives.
Recognition	Formalised CHW status and cohesion with facility teams.	• Create an official, criteria-based recognition system (such as certificates/medals awarded by local/state authorities). • Facilitate basic administrative procedures for CHWs (such as IDs, attestations, renewals). • Promote joint health facility–CHW team-building and routine inclusion in staff meetings.
Coordination	Alignment of partners and programmes	• Apply a “one approach, one budget, one plan” principle through the existing quarterly Commission for communal health development [locally: Commission Communale de Développement de la Santé (CCDS)]. • Define clear roles/responsibilities with local, multisectoral players, aligned with national guidance where it exists. • Co-develop a fixed monthly CHW activity calendar with health-facility leads. • Simplify and standardise reporting tools and flows; consider light digital tools where feasible and sustainable.
Training (Formation)	Continuous and integrated training	• Conduct regional training-needs diagnostics. • Deliver unified, interactive training modules, including TB and HIV, co-developed with national programmes. • Schedule biannual (semestrial) refreshers and formative follow-up approximately one month post-training. • Build a trainer pool at the health facility level and review training impact regularly.
Equipment & materials	Continuous availability of simple, functional equipment and tools	• Launch participatory inventories with local, district, and regional stakeholders. • Prioritise essential kits by daily utility and context. • Centralise storage at health facilities, trace deliveries electronically. • Plan regular inventories, annual impact reviews, and community-led maintenance (committees and small repair funds).
Supervision	Structured coaching and supportive supervision	• Institutionalise a “supportive supervision culture” (coaching-based, solution-oriented supervision). • Standardise checklists and integrate TB/HIV into supervision templates; digitise simplified reporting where connectivity allows. • Program quarterly field visits, piggybacking on advanced health facility outreach strategies to optimise travel and costs.
Security	Protective working environment	• Ensure continuous provision of basic Personal Protective Equipment (PPE). • Embed CHW safety in community charters (e.g., local *Dina Be* customary codes). • Establish emergency hotlines co-managed by MoH and partners. • Recruit CHWs from remote settlements where appropriate to improve reach to hard-to-access areas. • Establish a buddy system for longer trips and share itineraries in advance with community leaders. • Provide visibility equipment (reflective vests, headlamps).

The framework focuses on the foundational support functions that determine whether CHWs can perform across programmes, specifying what CHWs should receive and how they should be enabled to fulfil their role, rather than adding new disease-specific responsibilities. Actions under each lever include, for example, transparent compensation mechanisms (Protection); harmonised reporting and aligned local coordination (Coordination); standardised training modules covering TB, HIV, and other disease domains (Formation); and structured supportive supervision focused on coaching rather than reporting (Supervision). Priority actions for each lever are detailed in Table 1.

## 4 Discussion

This community case study presents the development of PROFESS, a CHW support framework co-designed by a cross-sector consortium in response to persistently low CHW engagement in TB and HIV care in Madagascar. Stage 1 combined quantitative evidence and qualitative accounts of the operational conditions constraining engagement, among others economic precarity among CHWs, inconsistent access to training and equipment, supervision focused on reporting rather than coaching, and limited recognition within communities and health actors. PROFESS, developed from the Stage 2 co-design process, translates the main support deficits identified in Stage 1, together with stakeholder priorities and recommendations, into seven operational levers with concrete actions.

Both the quantitative evidence and the co-design discussions highlighted that a central constraint on CHW engagement in TB and HIV is the overall vertical, topic-specific, and partial rather than comprehensive form of support provided by a multitude of stakeholders; a phenomenon commonly referred to as fragmentation. PROFESS does not, on its own, defragment the CHW support landscape, yet our case shows that a co-designed, ministry-validated support framework can specify operational responses to concrete deficits of underlying fragmentation, give it operational consequence, and support mutual accountability.

Several individual PROFESS levers also connect to established literature: the (financial) Protection lever aligns with growing recognition that what is often described as low CHW “motivation” is more accurately addressed as structural under-enablement (29); the Supervision lever aligns with evidence that supportive, coaching-based supervision outperforms inspection-focused models (30). Our case furthermore illustrates data and process fragmentation described by Ballard and colleagues, in which actors pursuing similar community-health goals use divergent indicators, definitions, and reporting processes (31). Overall, our case adds to existing work on harmonising CHW support (32, 33) by showing how broad principles of coordination, integration, and sustainability can be translated, through participatory co-design, into a concrete operational guidance.

### 4.1 Lessons learned and transferability

We learned two main lessons for strengthening CHW engagement and the underlying workforce-support architecture:

First, workforce-support frameworks designed within a single disease context can be transferable when anchored in foundational system functions. PROFESS was elaborated using TB and HIV as priority focus areas, but its seven levers are disease-agnostic and structurally adaptable. Frameworks built around system functions are therefore better candidates for cross-domain transfer than disease-specific protocols. However, transferability is not automatic but conditional on context assessment and deliberate adaptation. Settings considering PROFESS adaptation should, from our perspective, assess three dimensions:

1. *System fragmentation and financing structure*: is CHW support fragmented across programmes and funders? Does the government provide limited direct funding? Madagascar exemplifies both; by contrast, settings with more unified, centralised CHW programming may require different coordination approaches.
2. *CHW formalisation and employment status*: are CHWs predominantly volunteer or formally employed? PROFESS's Financial Protection and Recognition levers assume volunteer or informally positioned CHWs; in formally employed contexts, the emphasis would shift accordingly.
3. *Existing coordination mechanisms*: does a functional platform exist for multi-sectoral coordination at district or commune level? PROFESS is currently piloted in geographically limited settings across an NGO working in different health domains. Adaptation requires identifying similar or analogous mechanisms in other settings.

The principles embedded in PROFESS's seven levers are from our perspective transferable across settings and disease domains, some operational specifics however may not. For example, PROFESS proposes “biannual training refreshers”, but this may need to be different in other settings depending on accessibility and/or geographical dispersion). Similarly, what constitutes “adequate compensation” depends on local cost of living and policy contexts and must be paired with workforce costing analyses and financing negotiations. Prospective adopters should view PROFESS as a framework for adaptation rather than a blueprint for direct adoption.

Second, co-design of a conceptual framework may only generate adoptable outputs when paired with operational elaboration, which also prompted the Ministry validation of PROFESS. Co-design produces principled priorities grounded in stakeholder expertise and lived experience, but institutional adoption requires the additional step of translating these into actions specific enough for programmes to implement and partners to coordinate around. The distinction between framework development and operational elaboration is, from our experience, critical for other settings attempting similar health system strengthening efforts and may explain why many participatory policy processes produce endorsed documents that are not subsequently implemented.

Our insights and approach from Madagascar may transfer to other settings characterised by systemic fragmentation and partner-driven implementation. Evidence from Ethiopia's Health Extension Workers shows that fragmentation can persist even in more institutionalised CHW programmes, suggesting that the operational questions PROFESS addresses are not limited to volunteer-based or partner-dependent systems (34). However, actual transferability remains untested. Successful implementation of PROFESS in Madagascar (pilot underway, results expected 2027) will generate evidence on feasibility and effects in the origin context. Adaptation and pilot testing of PROFESS in ≥1 contrasting setting would further test whether the framework functions across different CHW employment models, financing structures, and disease burdens, illuminating which levers prove universally applicable and where context-specific modifications are essential.

### 4.2 Limitations and next steps

The co-design process faced process-related, feasibility, and methodological limitations. Some Ministry representatives attended only intermittently due to competing professional responsibilities; while their participation remained valuable, intermittent attendance illustrates a common challenge in engaging senior policy-level actors in intensive participatory processes. Another feasibility tension emerged during the workshop itself as several participant proposals, particularly those related to financial compensation and social protection, exceeded thresholds set within current national policy and financing frameworks. Ministry representatives recognised the importance and legitimacy of these proposals but expressed reservations about feasibility under current structural and financial constraints. This tension is instructive: participatory co-design can surface priorities grounded in frontline realities while making visible the policy, budgetary, and institutional constraints that shape what can be implemented in the short term. Methodologically, the workshop drew on one cohort of stakeholders with specific expertise in TB and HIV; perspectives from CHWs and partners in non-participating regions, and from stakeholders working in other disease domains, are not represented.

Two further gaps remain unresolved by PROFESS itself: neither national policy documents nor our framework provide guidance on geographic prioritisation (“where to work first”) or on sequencing across levers (“what to do first”), including a minimum viable support package for phased implementation.

A pilot implementation of a PROFESS-based intervention is underway to assess the feasibility and early effects on CHW engagement and service delivery, with first results expected by early 2027.

## 5 Conclusion

PROFESS translates foundational principles of CHW support into seven concrete operational levers, specifically tailored to the specific context of low CHW engagement in TB and HIV care in Madagascar. By focusing on foundational workforce-support functions rather than disease-specific protocols, PROFESS provides a transferable scaffold for strengthening CHW engagement across health domains. The validated Guide itself signals this transferability explicitly, extending its principles to other health domains and programmes. An ongoing pilot implementation will generate evidence on its effects on engagement and service delivery, as well as on the operational sequencing that PROFESS itself does not yet resolve and may inform similar efforts in other settings where workforce evidence is scarce and support fragmented.

## Data Availability

The original contributions presented in the study are included in the article/Supplementary Material, further inquiries can be directed to the corresponding author.
