# Correction: Co-designing PROFESS: a community health worker support framework to strengthen engagement in TB and HIV care in Madagascar

**DOI:** 10.3389/frhs.2026.2003133

**Published:** 2026-10-09

**Authors:** Frontiers Production Office

**Affiliations:** Frontiers Media SA, Lausanne, Switzerland

**Keywords:** co-design, community health workers, design thinking, health system fragmentation, implementation, service integration, TB/HIV

Affiliation ‘Life Member, Nepal Public Health Association, Nepal' for reviewer Gaurav Devkota was erroneously given as ‘World Health Organisation (Nepal), Nepal'.

The original version of this article has been updated.

