# Supplementary material for: Co-designing PROFESS: a community health worker support framework to strengthen engagement in TB and HIV care in Madagascar
Source: Front Health Serv. 2026 Sep 18;6:1910840. doi: 10.3389/frhs.2026.1910840 (PMC13631075; doi:10.3389/frhs.2026.1910840)
Supplement: Supplementary file 1 [file Datasheet1.pdf]

# Guide PROFESS

## Renforcer les Agents Communautaires dans la réponse à la tuberculose et au VIH à Madagascar: Sept leviers pour agir.

Recommandations issues d'un atelier design thinking

Madagascar, Mars 2025

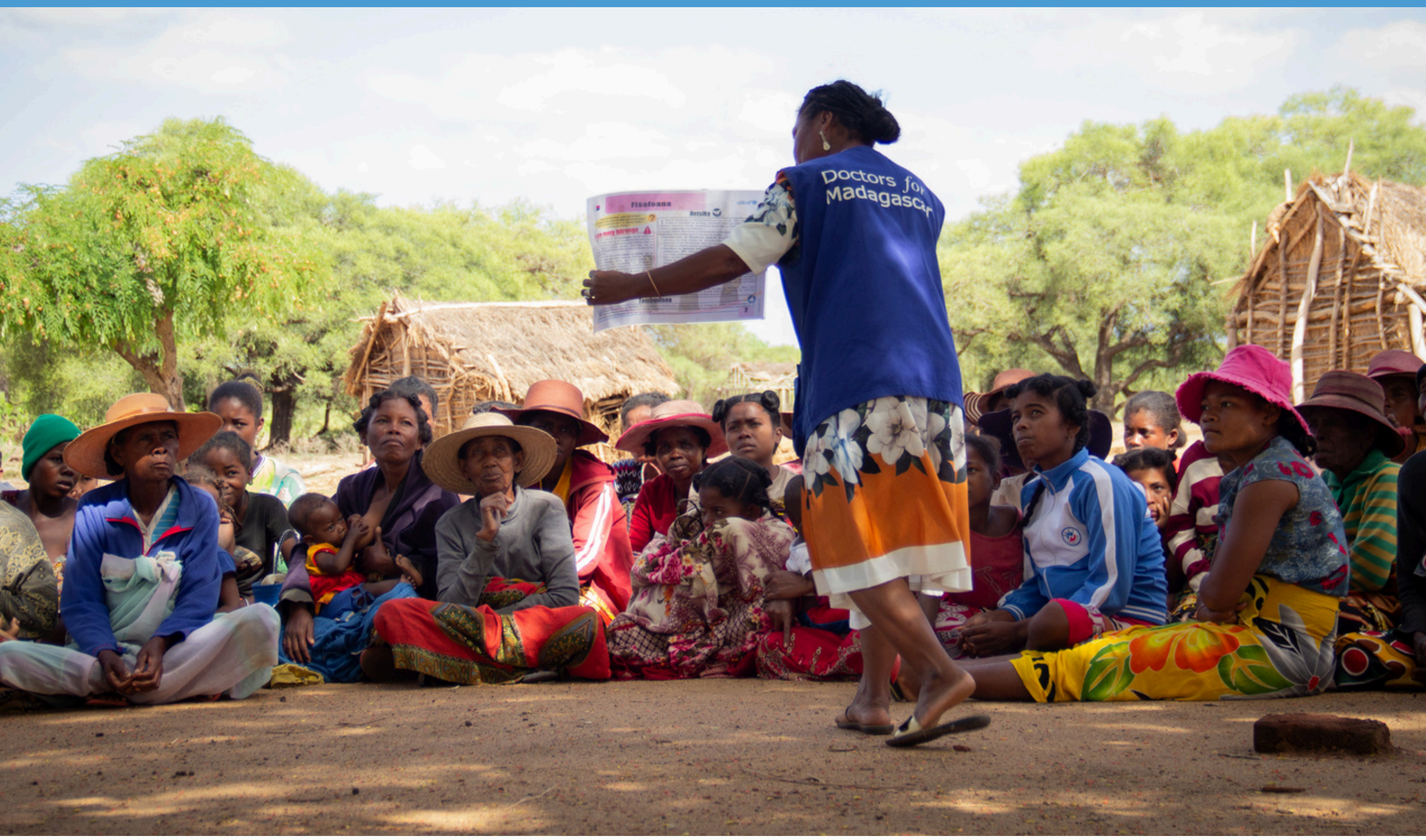

Ministeran'ny Fahasalamam-bahoaka

CHARITÉ UNIVERSITÄTSMEDIZIN BERLIN

Doctors for Madagascar

**Métadonnées recommandées :**
***Titre complet*** : Renforcer les Agents Communautaires dans la réponse à la tuberculose et au VIH à Madagascar : sept leviers pour agir. Guide « PROFESS » – Recommandations issues d'un atelier de design thinking, Madagascar, mars 2025
***Auteurs principaux avec organisations d'affiliation*** *:*
Onja Gabrielle Ravololohanitra - *Doctors for Madagascar*
Michel Harison Tiaray - *Programme national de lutte contre la tuberculose*
Norosoa Assunta Felananirina Rakotoherimora - *Programme national de lutte contre les IST et le Sida*
Haingotiana Razafimanantsoa - *Doctors for Madagascar*
Larissa Hecht - *Charité – Universitätsmedizin Berlin*
Nadine Muller - *Charité – Universitätsmedizin Berlin*
***Date de publication*** : 2026
***Lieu*** : Antananarivo, Madagascar
**Version** : Version finale – mars 2026
***Mots-clés***: Agents communautaires; tuberculose; VIH; Madagascar; Design thinking; PROFESS
***Suggestion de citation***: Ravololohanitra OG, Tiaray MH, Rakotoherimora NAF, Razafimanantsoa H, Hecht L, Muller N. *Renforcer les Agents Communautaires dans la réponse à la TB et au VIH à Madagascar : sept leviers pour agir. Guide « PROFESS ». Recommandations issues d'un atelier de design thinking, Madagascar, mars 2025*. Antananarivo: Doctors for Madagascar; Charité – Universitätsmedizin Berlin; Programme national de lutte contre la tuberculose; Programme national de lutte contre les IST et le Sida; 2025.
***Bailleurs de fonds***: Else Kröner-Fresenius-Stiftung (EKFS); Berlin Institute of Health (BIH).
***Déclaration d'indépendance éditoriale***: Le contenu de ce document relève de la seule responsabilité de ses auteurs et des organisations rédactrices. Les bailleurs n'ont exercé aucune influence sur l'élaboration, l'analyse ni les conclusions présentées.

# Synthèse exécutive

À Madagascar, moins de 5 % des agents communautaires (AC) participent actuellement à la riposte à la TB et le VIH, faute de statut clair, de ressources adéquates et de coordination. Un atelier de design thinking tenu en mars 2025, réunissant des acteurs de différents niveaux (AC, ONG, programmes nationaux et autorités), a dégagé sept leviers stratégiques synthétisés dans l'acronyme **PROFESS**: **P**rotection financière, **R**econnaissance, co**O**rdination, **F**ormation, **É**quipement, **S**upervision et **S**écurité. Le présent guide détaille ces recommandations afin de renforcer l'implication des AC dans la lutte contre les deux maladies. Leur mise en œuvre concertée, adossée à un financement durable, professionnalise les AC, accélère le dépistage et rapproche la couverture sanitaire universelle.

# Cadre PROFESS

**Protection financière**

- Mise en place d'une indemnisation transparente, régulière et proportionnelle
- Appui à la couverture de santé
- Appui à la génération de revenus

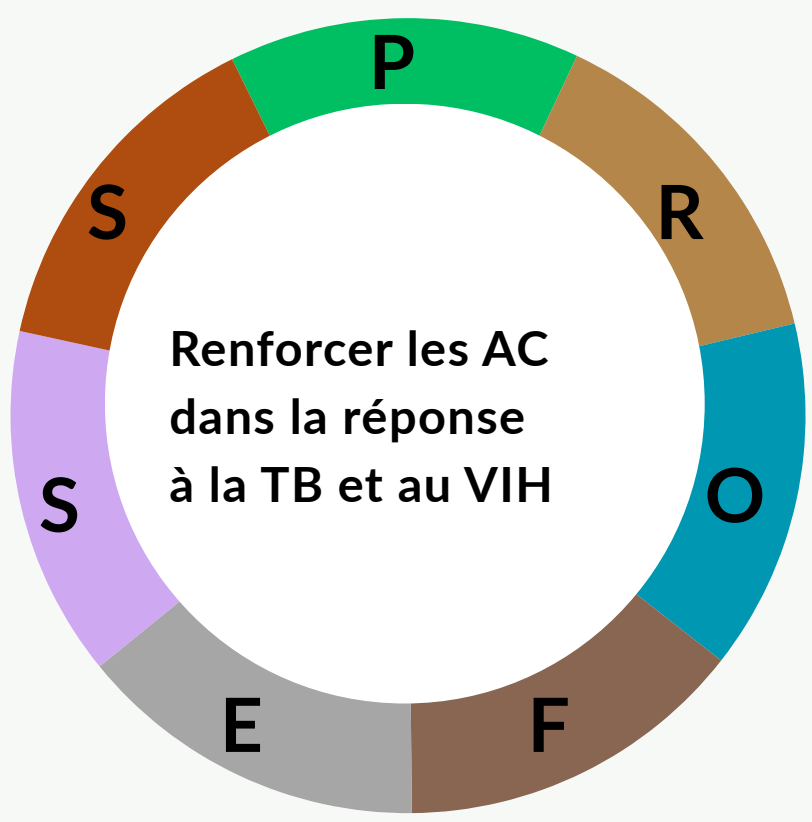


**Reconnaissance**

- Mise en place d'un système officiel de reconnaissance par les autorités locales
- Implication communautaire dans la valorisation des AC.
- Promotion de la cohésion du personnel des CSB et des AC.

**Coordination**

- Renforcement de la coordination multisectorielle.
- Utilisation de la réunion trimestrielle du CCDS (Commission Communale de Développement Sanitaire).
- Présentation et validation des plans d'action des agents communautaires lors de ces réunions
- Clarification des rôles et responsabilités des agents communautaires.
- Alignement de toutes les activités des agents communautaires sur les lignes directrices nationales de la santé communautaire.
- Mise en place ou renforcement d'un système de rapportage simple et harmonisé pour les agents communautaires.

**Formation**

- Mise en place d'un planning de formations régulières et continues.
- Assurance d'accès aux formations pour tous les AC, y compris en zones reculées.
- Adaptation du contenu de formation aux thèmes pertinents (TB, VIH, communication, etc.).
- Évaluation et suivi de l'impact des formations..

**Equipements et matériel**

- Mise à disposition des outils simples, fonctionnels et adaptés au contexte local.
- Distribution régulière et équitable des équipements et matériels nécessaires.
- Maintenance et remplacement des équipements usés.

**Supervision**

- Mise en place d'un système de supervision bienveillant et structuré.
- Assurance de visites régulières de terrain des superviseurs.
- Formation des superviseurs en feedback constructif et personnalisé.
- Renforcement du lien entre superviseurs et AC.

**Sécurité**

- Assurance de l'accès aux équipements de protection individuelle.
- Renforcement de la sécurité physique des AC lors des déplacements, incluant les risques environnementaux et sociaux.
- Soutien en cas d'incident ou de menace.

# Glossaire

**AGR** : Activités génératrices de revenus
**AC** : Agents communautaires
**ASOS** : Action Socio-Sanitaire Organisation Secours
**CCDS** : Commission Communale de Développement de la Santé
**CDT** : Centre de diagnostique et de traitement
**CSB** : Centre de santé de base
**DfM** : Doctors for Madagascar
**DLMT** : Direction de Lutte contre les Maladies Transmissibles
**DPS** : Direction de la Promotion de la Santé
**DSSB** : Direction des Soins de Santé de Base
**EMAD** : Équipe de management du district
**EMAR** : Équipe de management de la région
**EPI** : Équipements de protection individuelle
**IST** : Infections sexuellement transmissibles
**ONG** : Organisation non gouvernementale
**PNLIST** : Programme National de Lutte contre les IST/SIDA
**PNLT** : Programme National de Lutte contre la Tuberculose
**PSI** : Population Services International
**PTF** : Partenaires techniques et financiers
**SAF/FJKM** : Sampan'Asa Fampandrosoana/ Fiangonan' i Jesoa Kristy eto Madagasikara (Département pour le Développement de l'église FJKM)
**SALFA** : Sampan'Asa Loterana momba ny FAhasalamana (Département de la santé de l'église Luthérienne malgache)
**TB** : tuberculose

# Contexte

Présents dans plus de 100 pays, les agents communautaires (AC) sont devenus un maillon essentiel des systèmes de santé. Véritables intermédiaires entre les structures de soins formelles et les communautés, ils constituent souvent le seul point d'accès aux soins en zone rurale, situation encore plus cruciale dans les pays à faible revenu comme Madagascar. Dans ce pays majoritairement rural, la pauvreté limite fortement l'accès à des services de santé de qualité. Parallèlement, la tuberculose (TB) et le VIH affichent toujours des taux de prévalence et une charge de morbidité élevés, voire croissants, aggravés par la stigmatisation qui freine le dépistage et le traitement.

Le Ministère chargé de la Santé Publique estime que moins de 5 % des AC à Madagascar participent aux activités de lutte contre la TB et le VIH. Cette faible implication sur le paquet d'activités spécifiques s'explique notamment par l'absence de stratégie nationale définissant clairement leur rôle, de directives sur leur formation et l'équipement nécessaire, le manque de mécanisme de soutien financier adapté ainsi que d'autres obstacles systémiques.

Pour relever ce défi, l'ONG Doctors for Madagascar et son partenaire académique, la Charité – Universitätsmedizin Berlin, ont réuni 37 acteurs de la santé communautaire, incluant des AC, d'autres intervenants de terrain ainsi que des représentants d'ONG et des responsables ministériels lors d'un atelier présentiel en mars 2025. Ensemble, ils ont identifié et élaboré des solutions adaptées, réalisables et immédiatement applicables pour renforcer l'implication et l'impact des AC dans la lutte contre la TB et le VIH.

# Objectif

Ce document a pour but de présenter les recommandations élaborées à l'issue de cet atelier d'experts et constitue ainsi un guide dans la mise en place de solutions adaptées au contexte local visant à renforcer l'implication et l'impact des AC dans la lutte contre la TB et le VIH à Madagascar.

# Méthodologie de l’atelier

Un atelier présentiel de trois jours, basé sur l’approche design thinking, a été organisé pour élaborer une stratégie opérationnelle visant à renforcer l’implication et l’impact des AC dans la lutte contre le VIH et la TB. L’événement a réuni AC, encadreurs et personnels de santé des régions Analamanga, Itasy et Atsimo Andrefana, ainsi que des représentants d’ONG (Doctors for Madagascar, PSI, SALFA, ASOS, SAF/FJKM), des équipes EMAD et EMAR d’Itasy et d’Atsimo Andrefana, et des représentants de la DPS, DSSB, DLMT, PNLIST et PNLT.

La méthode visait à stimuler la créativité, favoriser l’engagement et garantir la participation de tous, quel que soit le niveau ou le secteur. Le programme alternait présentations, travaux de groupe, exercices individuels et discussions plénières. Les groupes, composés à l’avance pour une représentation équilibrée, encourageaient la libre expression.

Une attention particulière a été accordée aux AC, issus de contextes variés, invités à partager leurs expériences, conditions de travail et contraintes. Leur plaidoyer a été appuyé par des données probantes issues d’une étude à méthodes mixtes menée par DfM et la Charité Berlin, offrant une vision claire de leurs caractéristiques socio-économiques, motivations, obstacles et défis.

L’atelier a été conçu autour des trois phases design thinking suivantes :
***Empathie & définition du problème*** : Les participants se sont mis dans la peau d’un AC pour inventorier tâches, outils, réussites et obstacles quotidiens.
***Idéation*** : Un large éventail de pistes a émergé grâce aux idées et retours de chacun.
***Élaboration*** : Les propositions retenues ont été transformées en actions précises, réalisables et adaptées au contexte.
En plaçant les AC au cœur du processus, l’atelier a fait émerger des solutions concrètes, adaptées et applicables, plutôt que de simples recommandations théoriques.

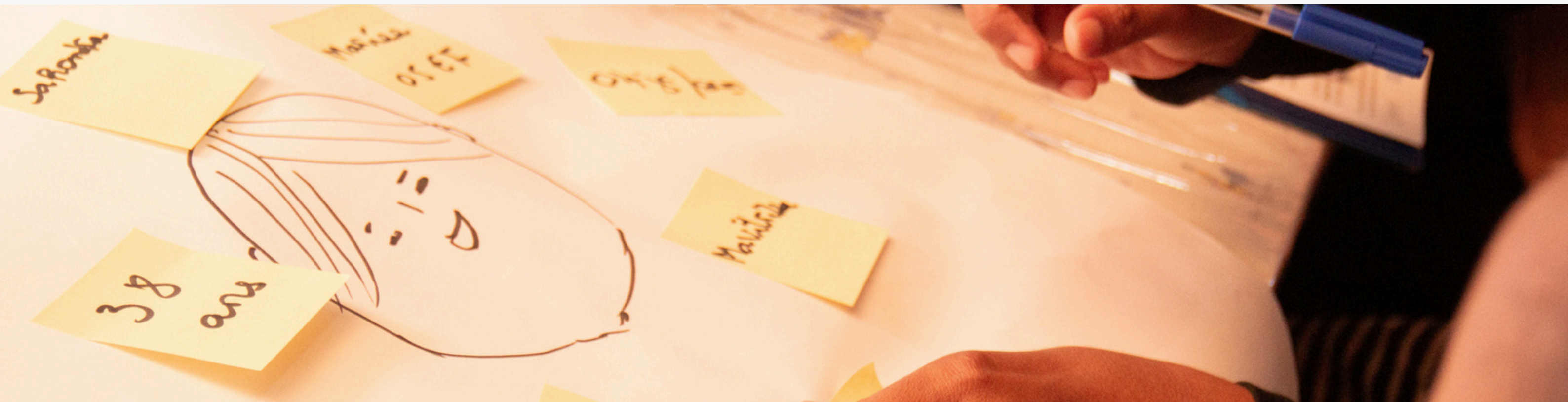

## Les sept leviers

L'atelier a dégagé sept leviers essentiels pour renforcer l'action des AC contre la TB et le VIH à Madagascar. Ils se résument dans l'acronyme PROFESS où chaque lettre correspond à un thème détaillé dans le tableau ci-après :

| | ***Leviers & objectifs*** | ***Éléments recommandés*** |
|---|---|---|
| **P** | **Protection financière**<br>*Assurer une compensation équitable et durable pour les AC* | • Mise en place d'une indemnisation transparente, régulière et proportionnelle.<br>• Appui à la couverture de santé.<br>• Appui à la génération de revenus. |
| **R** | **Reconnaissance**<br>*Valoriser le rôle des AC* | • Mise en place d'un système officiel de reconnaissance par les autorités locales.<br>• Implication communautaire dans la valorisation des AC.<br>• Promotion de la cohésion du personnel des CSB et des AC. |
| **O** | **Coordination**<br>*Harmoniser les interventions autour des AC* | • Renforcement de la coordination multisectorielle.<br>• Utilisation de la réunion trimestrielle du CCDS. (Commission Communale de Développement Sanitaire)<br>• Présentation et validation des plans d'action des agents communautaires lors de ces réunions.<br>• Clarification des rôles et responsabilités des agents communautaires.<br>• Alignement de toutes les activités des agents communautaires sur les lignes directrices nationales de la santé communautaire.<br>• Mise en place ou renforcement d'un système de rapportage simple et harmonisé pour les agents communautaires. |
| **F** | **Formation**<br>*Renforcer les compétences des AC* | • Mise en place d'un planning de formations régulières et continues.<br>• Assurance d'accès aux formations pour tous les AC, y compris en zones reculées.<br>• Adaptation du contenu de formation aux thèmes pertinents (TB, VIH, communication, etc.) Évaluation et suivi de l'impact des formations. |

| | | |
|---|---|---|
| **E** | **Équipements et matériels**<br>*Garantir les équipements et matériels nécessaires aux AC* | • Mise à disposition des outils simples, fonctionnels et adaptés au contexte local.<br>• Distribution régulière et équitable des équipements et matériels nécessaires.<br>• Maintenance et remplacement des équipements usés. |
| **S** | **Supervision**<br>*Accompagner constructivement la performance des AC* | • Mise en place d'un système de supervision bienveillant et structuré.<br>• Planification de visites régulières de terrain des superviseurs.<br>• Formation des superviseurs en feedback constructif et personnalisé.<br>• Renforcement du lien entre superviseurs et AC. |
| **S** | **Sécurité**<br>*Protéger les AC dans leur fonction* | • Accès garanti aux équipements de protection individuelle.<br>• Renforcement de la sécurité physique des AC lors des déplacements, incluant la protection contre les risques environnementaux et sociaux.<br>• Soutien en cas d'incident ou de menace. |

# Recommandations opérationnelles

| P | Protection financière |
|---|---|

**Défis**

- Indemnités trop faibles et versées de façon irrégulière
- Dépenses personnelles non remboursées

**Objectifs**

1. Ajuster les indemnités à la charge réelle de travail.
2. Assurer un paiement régulier et transparent.
3. Offrir une couverture sociale de base aux AC et à leur famille.
4. Renforcer les activités génératrices de revenus (AGR).

**Actions**

***P1 : Révision du système d'indemnisation***

● Recenser tous les AC, incluant leurs coordonnées de contact, cartographie des activités, implications réelles, formations et équipements reçus, ONG collaboratrices.

● Définir une grille d'indemnités :

○ **Allocation fixe**, avec activités attendues clairement définies : 50 000 Ar par mois

○ **Remboursement de frais de transport** si non inclus dans l'allocation fixe (p.ex. 100 km × 500 Ar/km) et d'autres dépenses personnelles (p.ex. achat des EPI, déplacement, référencement des malades, crédits de communication).

○ **Prime de performance** si pas couverte par l'allocation fixe (p.ex. 2 000 à 10 000 Ar/cas dépisté)

○ **Indemnités de participation aux formations** harmonisées avec ceux mentionnés par le guide MEO: Hébergement hors fokontany à 10.000 Ariary, Indemnité journalière 15.000 Ariary.

● Élaborer et distribuer des fiches techniques par type d'indemnisation détaillant clairement les critères, modalités, fréquences, et montants.

● Mener une concertation avec la DSSB pour validation des grilles proposées.

***P2 : Sécurisation des paiements***

● Choisir avec les AC concernés le mode de versement (Mobile Money, espèces) en tenant compte des contraintes d'accessibilité au réseau.

● Tracer et justifier les transactions via un outil numérique ou registre simple et transparent.

● Former les AC et les superviseurs à l'utilisation de l'outil.

● Établir et publier un calendrier mensuel de paiement qui garantit la régularité des paiements, par la fixation de dates précises chaque mois, la désignation de responsables chargés du processus, incluant une stratégie de suivi et évaluation claire.

***P3 : Couverture santé des AC et de leurs familles***

● **Sonder les besoins** en couverture santé des AC concernés, incluant maladies fréquentes, dépenses effectuées, vulnérabilités.

● Mettre en place un **système de protection sociale ou d'assurance maladie** (p.ex. cotisations, mutuelles, subventions, fonds d'équité).

● Négocier les possibilités d'adhésion à une **mutuelle ou dans les dispositifs existants.**

| P | Protection financière |
| --- | --- |

***P4 : Soutien aux AGR***

- Appuyer à créer des **associations d'AC** par CSB (p.ex. appui à la structuration, élection des membres, statut enregistrement administratif).
- Former les associations à la **gestion de micro-projets.**
- Fournir des **kits pour les AGR** selon le type d'activité économique familiale ou de l'association des AC.

***P5 : Scolarité gratuite***

- Plaidoyer pour conclure un accord interministériel pour exonérer les frais d'inscription des enfants des AC.

| R | Reconnaissance |
| --- | --- |
| **Défis**<br>• Absence de cadre officiel de valorisation<br>• Reconnaissance informelle peu motivante | **Objectifs**<br>1. Instaurer une reconnaissance officielle de l'État.<br>2. Célébrer publiquement les AC méritants.<br>3. Renforcer la cohésion entre AC et personnel de santé. |

## Actions

***R1 : Système de distinction nationale***

● Élaborer un **cadre formel de reconnaissance** (p.ex. certificat ou médaille honorifique) attribué par l'État ou les autorités sanitaires locales.

● Définir des critères de sélection tout en considérant les indicateurs dans le manuel de motivation des AC.

● **Impliquer les autorités locales et sanitaires** et le **CCDS** dans le processus de sélection et de remise.

● Organiser des **cérémonies publiques** de remise de médailles ou certificats à l'occasion d'événements sanitaires (journée mondiale de la santé, etc.) pour ajouter du poids à la reconnaissance donnée.

***R2 : Facilitation des procédures administratives***

Faciliter l'obtention des papiers administratifs au niveau des centres administratifs locaux pour les AC et leurs familles (p.ex. copie d'acte de naissance, certificat de résidence).

***R3 : Promotion de la cohésion***

Cohésion entre AC et personnel de santé à travers des activités de team building ou repas en commun.

| O | CoOrdination |
| --- | --- |

| Défis | Objectifs |
| --- | --- |
| • Rôles des acteurs mal définis<br>• Coordination fragmentée<br>• Outils de reporting complexes | 1. Mettre à jour des lignes directrices.<br>2. Simplifier le système de reporting.<br>3. Promouvoir la coordination multisectorielle.<br>4. Partager des plans d'actions entre toutes les parties prenantes. |

**Actions**

***O1 : Principe "One approach - one budget - one plan"***

**Utiliser les réunions trimestrielles du CCDS** (Commission Communale de Développement de la Santé) comme principal mécanisme de **coordination multisectorielle**, impliquant les AC, les agents de santé, les autorités locales et les PTF.

***O2 : Plan d'actions***

Pendant le regroupement mensuel, les chefs CSB accompagnent les AC dans l'établissement de leur plan d'actions.

***O3 : Reporting digitalisé***

Mettre en place un **système de reportage électronique** adapté au contexte local en collaboration avec les structures de santé.

| F | Formation |
|---|---|

| Défis | Objectifs |
|---|---|
| • Contenus peu adaptés au contexte<br>• Manque de formateurs locaux | 1. Adapter les modules aux besoins régionaux.<br>2. Créer un vivier de formateurs au niveau CSB.<br>3. Intégrer TB/VIH dans un module unique.<br>4. Assurer un recyclage trimestriel. |

**Actions**

***F1 : Diagnostic des besoins***

● Mener, région par région, une **analyse détaillée des besoins de formation** des AC.
● **Adapter les modules de formation** en conséquence en fonction des besoins identifiés.

***F2 : Module TB/VIH intégré***

● Concevoir un **module unique TB/VIH** en collaboration avec le PNLIST et le PNLT.
● Organiser des sessions en présentiel, interactives, axées sur le partage d'expériences et le réseautage.
● Prévoir un suivi formatif un mois après la formation et un recyclage semestriel.
● Planifier les formations par axe sanitaire durant la saison sèche.
● Réaliser une supervision formative au cours du regroupement mensuel et lors des supervisions sur site.

***F3 : Formation des formateurs***

● **Former en priorité les chefs CDT et les formateurs des CSB**, tant sur les contenus de base que sur les thèmes spécifiques, afin qu'ils puissent assurer le transfert de ces connaissances auprès des AC.

***F4 : Suivi d'impact***

● Réunir régulièrement les chefs CDT ou responsables CSB pour **évaluer l'impact de la formation** sur la performance des AC.
● Effectuer des **supervisions pratiques sur site** pour consolider les acquis et renforcer durablement les compétences.

| E | Équipements et matériels |
|---|---|

| Défis | Objectifs |
|---|---|
| • Dotations insuffisantes ou mal réparties<br>• Entretien négligé | 1. Établir un registre détaillé des besoins.<br>2. Garantir une dotation équitable et suivie.<br>3. Mettre en place un plan de maintenance participatif. |

**Actions**

***E1 : Inventaire et priorisation***

● Réaliser, région par région, un **inventaire participatif des matériels existants et des besoins**, avec la contribution des AC, chefs CSB, EMAD et EMAR.
● **Classer et hiérarchiser les équipements** en fonction de l'utilité quotidienne des AC et du contexte local.
● Négocier la **couverture de tous les AC** à travers la synthèse des besoins avec les PTF par l'intermédiaire du PNLT et du PNLIST.

***E2 : Logistique et traçabilité***

● Définir, avec les PTF, PNLIST, PNLT, EMAR et EMAD, une **liste d'équipements** essentiels et un **calendrier de livraison**.
● Centraliser le **stockage au niveau des CSB et déployer un registre électronique** pour tracer réception et distribution
● Programmer un **inventaire semestriel** et une **évaluation annuelle** de l'impact du matériel sur la performance des AC, en collaboration avec les CSB.

***E3 : Entretien communautaire***

● Sensibiliser et impliquer la communauté (travaux de réparation, nettoyage périodique).
● Créer des **comités locaux de suivi du matériel**.
● Mobiliser un **appui financier pour la maintenance et le remplacement** des pièces.

| S | Supervision |
|---|---|

| Défis | Objectifs |
|---|---|
| • Visites rares et perçues comme “punitives”<br>• Manque de ressources et d’outils | 1. Promouvoir une supervision formative et bienveillante.<br>2. Standardiser et digitaliser les outils.<br>3. Inclure systématiquement TB/VIH. |

**Actions**

***Su1 : Culture de bienveillance***

● Instaurer une **supervision formative centrée sur le soutien** plutôt que le contrôle.

● Expliquer aux AC les objectifs réels et les bénéfices concrets de la supervision.

***Su2 : Visites de terrain***

● Programmer, chaque trimestre, des **visites de supervision sur le terrain en profitant des stratégies avancées** menées par les chefs CSB **pour mutualiser les déplacements**.

● **Intégrer systématiquement les volets TB et VIH** dans les canevas de supervision existants.

***Su3 : Outils standardisés***

● **S’appuyer sur les outils existants**: entre autres, grille de supervision de routine des AC.

● Développer des outils de reporting simplifiés et, si possible, numérisés, **en complément des canevas officiels**, compatibles avec la connectivité locale.

| S | Sécurité |
|---|---|

| Défis | Objectifs |
|---|---|
| • Déplacements longs, parfois nocturnes<br>• Équipements de protection limités | 1. Réduire les risques liés aux trajets.<br>2. Mettre en place le travail en binôme.<br>3. Mobiliser les autorités locales pour la protection des AC. |

**Actions**

***Sé1 : Diminuer les trajets***

● Recruter en priorité des **AC domiciliés dans les zones d'intervention**, surtout dans les localités les plus éloignées, afin de limiter les distances à parcourir.

***Sé2 : Organisation et EPI***

● Instaurer un **système de binôme** obligatoire pour tout déplacement long, piloté par le chef CSB.
● Élaborer, avec les AC, un **planning de déplacements** communiqué à l'avance aux responsables communautaires.
● Fournir un **kit complet de protection et de visibilité** (gilet fluorescent, lampe frontale).

***Sé3 : Soutien des autorités***

● Intégrer la **sécurité des AC dans la charte communautaire « Dina Be »**, en collaboration avec les maires, chefs de fokontany et leaders religieux/traditionnels.
● Mettre en place une **ligne verte d'urgence**, gérée conjointement par le Ministère de la Santé Publique et les PTF.

# Agir ensemble

Le guide **PROFESS** offre une feuille de route claire ; sa réussite dépend d'un engagement collectif et coordonné. Il ne vise ni à créer une fragmentation supplémentaire, ni à restreindre son utilisation au seul renforcement de l'engagement des AC dans la réponse à la TB et au VIH : ses principes sont pleinement transférables à d'autres domaines de santé (vaccination, santé maternelle et infantile, etc.) ainsi qu'à d'autres profils communautaires nécessitant un renforcement structuré, tels que les éducateurs pairs.
Nous appelons tous les acteurs impliqués dans la santé communautaire à Madagascar à :

- **Instituer** : adopter officiellement PROFESS comme référence nationale et l'intégrer dans les politiques TB/VIH ainsi que dans d'autres programmes de santé prioritaires.
- **Financer** : sécuriser des ressources pour la rémunération, la formation continue, les équipements essentiels et la supervision digitale.
- **Mettre en œuvre** : traduire chaque levier en actions trimestrielles simples (paiements ponctuels, modules TB/VIH unifiés, reporting allégé, EPI disponibles, etc.) et les décliner pour d'autres interventions communautaires.
- **Suivre et ajuster** : utiliser des indicateurs communs, tels que la couverture AC, les délais diagnostiques, la satisfaction des AC, et réviser les stratégies à partir des données représentatives du terrain, en favorisant l'harmonisation inter-programmes.
- **Valoriser** : reconnaître publiquement les AC, garantir leur sécurité et favoriser la participation des communautés à l'entretien des équipements.

En s'inspirant de PROFESS et en collaborant étroitement, les acteurs pourront bâtir un système de soins communautaires véritablement intégral, au bénéfice de toutes les populations malgaches.

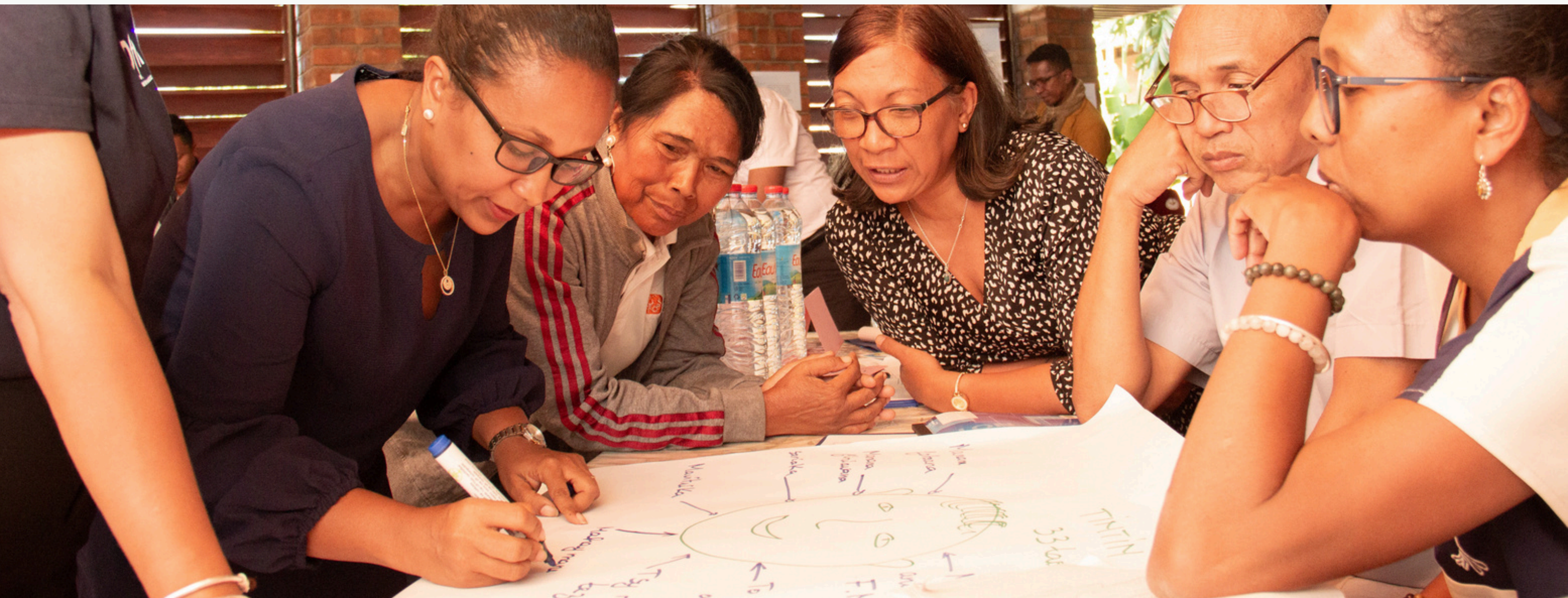

# Guide PROFESS

## Renforcer les Agents Communautaires dans la réponse à la tuberculose et au VIH à Madagascar: Sept leviers pour agir.

Recommandations issues d'un atelier design thinking

Madagascar, Mars 2025

**Auteurs principaux :** Onja Gabrielle **Ravololohanitra**, Michel Harison **Tiaray**, Norosoa Assunta **Rakotoherimora**, Haingotiana **Razafimanantsoa**, Larissa **Hecht**, Nadine **Muller**
