# Supplementary material for: Co-designing PROFESS: a community health worker support framework to strengthen engagement in TB and HIV care in Madagascar
Source: Front Health Serv. 2026 Sep 18;6:1910840. doi: 10.3389/frhs.2026.1910840 (PMC13631075; doi:10.3389/frhs.2026.1910840)
Supplement: Supplementary file 2 [file Datasheet2.pdf]

PROFESS Guide

Strengthening community health workers in the response to tuberculosis and HIV in Madagascar: seven levers for action

Recommendations from a design thinking workshop

Madagascar, March 2025

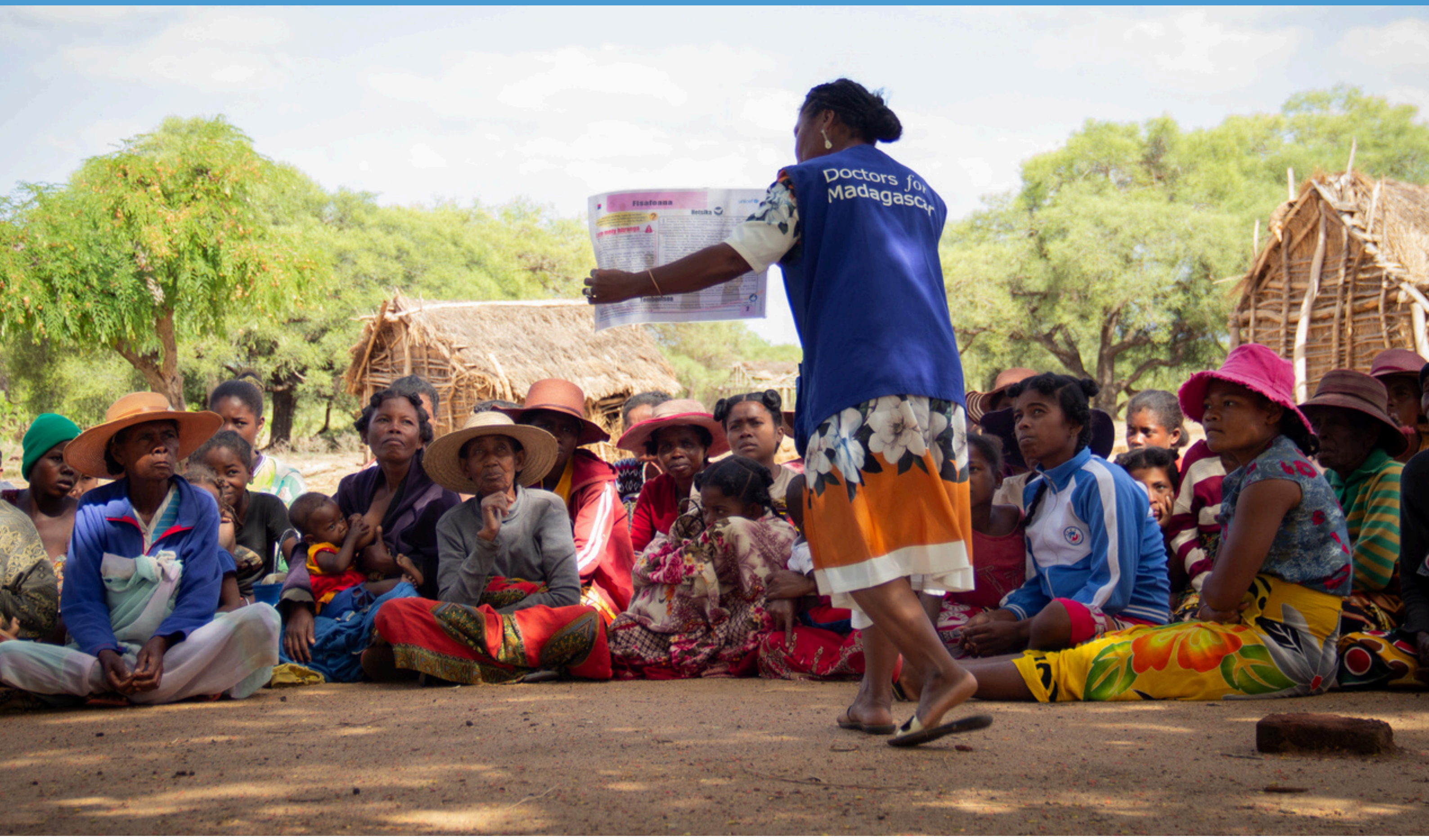

Ministeran'ny Fahasalamam-bahoaka

CHARITÉ UNIVERSITÄTSMEDIZIN BERLIN

Doctors for Madagascar

Recommended metadata:

Complete title: Strengthening community health workers in the response to tuberculosis and HIV in Madagascar: seven levers for action. “PROFESS” Guide – Recommendations from a design thinking workshop, Madagascar, March 2025

Lead authors with affiliated organizations:

Onja Gabrielle Ravololohanitra - *Doctors for Madagascar*

Michel Harison Tiaray - *Programme national de lutte contre la Tuberculose*

Norosoa Assunta Felananirina Rakotoherimora - *Programme national de lutte contre les IST et le Sida*

Haingotiana Razafimanantsoa - *Doctors for Madagascar*

Larissa Hecht - *Charité – Universitätsmedizin Berlin*

Nadine Muller - *Charité – Universitätsmedizin Berlin*

Publication date: 2026

Location: Antananarivo, Madagascar

Version: Final version – March 2026

Key words: Community health workers; Tuberculosis; HIV; Madagascar; Design thinking; PROFESS

Suggested citation: Ravololohanitra OG, Tiaray MH, Rakotoherimora NAF, Razafimanantsoa H, Hecht L, Muller N. *Strengthening Community Health Workers in the Response to Tuberculosis and HIV in Madagascar: Seven Levers for Action. “PROFESS” Guide. Recommendations from a design thinking workshop, Madagascar, March 2025*. Antananarivo: Doctors for Madagascar; Charité – Universitätsmedizin Berlin; Programme national de lutte contre la Tuberculose; Programme national de lutte contre les IST et le Sida; 2025.

Funders: Else Kröner-Fresenius-Stiftung (EKFS); Berlin Institute of Health (BIH)

Declaration of editorial independence: The content of this document is the sole responsibility of its authors and the organizations that drafted it. The donors had no influence on its development, analysis, or conclusions.

Executive Summary

In Madagascar, fewer than 5% of community health workers (CHWs) are currently involved in the response to tuberculosis (TB) and HIV, due to the lack of clear status, adequate resources, and coordination. A design thinking workshop held in March 2025 brought together actors from different levels, including CHWs, non-governmental organizations (NGOs), national programs and authorities, and identified seven strategic levers summarized in the acronym **PROFESS**: Financial **P**rotection, **R**ecognition, co**O**rdination, **F**ormation (Training), **E**quipment, **S**upervision and **S**ecurity. This guide details these recommendations in order to strengthen the involvement of CHWs in the fight against the two diseases. Their concerted implementation, backed by sustainable financing, supports CHW professionalization, speeds up case detection, and brings universal health coverage closer.

PROFESS framework

Financial Protection

- Establishment of transparent, regular, and proportional compensation
- Support for health coverage
- Support for income generation

Security

- Ensuring access to personal protective equipment
- Enhancing the physical security of CHWs when traveling, including environmental and social risks
- Support in the event of an incident or threat

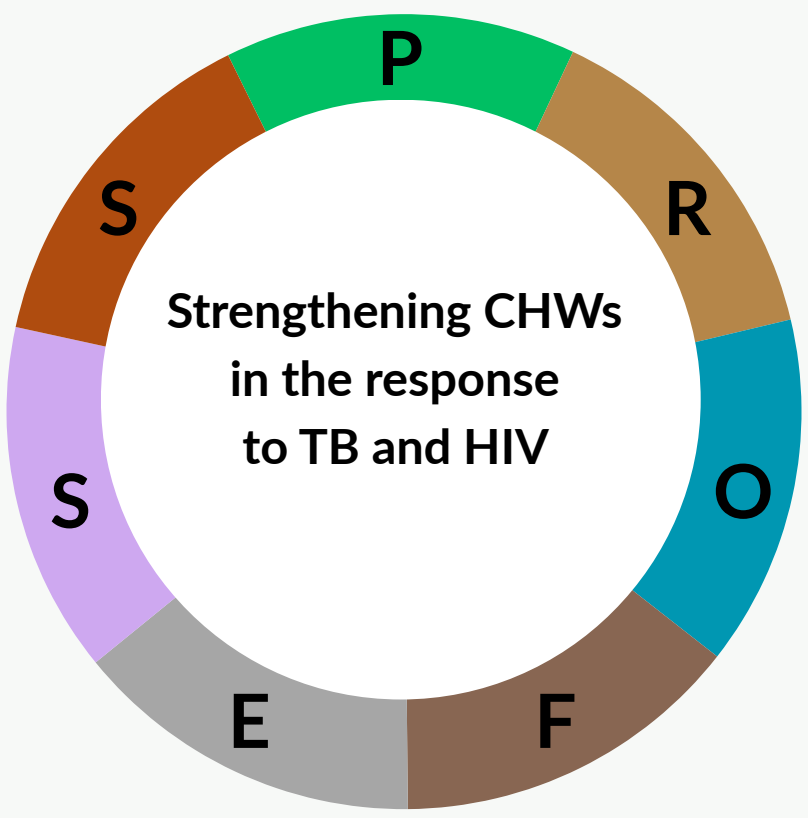

Recognition

- Establishment of an official recognition system by local authorities
- Community involvement in promoting CHWs
- Promotion of cohesion among CSB and CHW staff

Supervision

- Implementation of a supportive and structured supervision system
- Regular field visits by supervisors
- Training supervisors in constructive and personalized feedback
- Strengthening the relationship between supervisors and CHWs

Coordination

- Strengthening multisectoral coordination.
- Utilizing the quarterly meetings of the CCDS.
- Presenting and validating community health workers' action plans during these meetings.
- Clarifying the roles and responsibilities of CHWs.
- Aligning all activities with national guidelines.
- Establishing a simple, shared reporting system for CHWs.

Equipment

- Provision of simple, functional tools adapted to the local context
- Regular and equitable distribution of necessary equipment and materials
- Maintenance and replacement of worn equipment

Formation (Training)

- Establishment of a schedule for regular and ongoing training
- Ensuring access to training for all CHWs, including those in remote areas
- Adaptation of training content to relevant topics (TB, HIV, communication, etc.)
- Evaluation and monitoring of the impact of training

Abbreviations

- **ASOS**: Action Socio-Sanitaire Organisation Secours *(Socio-Health Action Relief Organization)*
- **CCDS** : Commission Communale de Développement de la Santé *(Municipal Health Development Commission)*
- **CDT**: Centre de diagnostique et de traitement *(Diagnosis and treatment center)*
- **CHW**: Community health worker
- **CSB**: Centre de santé de base *(Primary healthcare facility)*
- **DFM**: Doctors for Madagascar
- **DLMT**: Direction de Lutte contre les Maladies Transmissibles *(Directorate for the Control of Communicable Diseases)*
- **DPS**: Direction de la Promotion de la Santé *(Directorate for Health Promotion)*
- **DSSB**: Direction des Soins de Santé de Base *(Directorate for Primary Health Care)*
- **EMAD**: Équipe de management du district *(District management team)*
- **EMAR**: Équipe de management de la région *(Regional management team)*
- **IGA**: Income generating activities
- **NGO**: Non-governmental organization
- **PNLIST**: Programme National de Lutte contre les IST/SIDA *(National Program for the fight against STIs/HIV & AIDS)*
- **PNLT**: Programme National de Lutte contre la Tuberculose *(National Tuberculosis Program)*
- **PPE**: Personal protective equipment
- **PSI**: Population Services International
- **SAF/FJKM:** Sampan'Asa Fampandrosoana / Fiangonan' i Jesoa Kristy eto Madagasikara *(Development department of the church of Jesus Christ in Madagascar (FJKM))*
- **SALFA**: Sampan'Asa Loterana momba ny FAhasalamana *(Health department of the Malagasy lutheran church)*
- **STI**: Sexually transmitted infection
- **TB**: Tuberculosis
- **TFP**: Technical and financial partners

Context

Present in more than 100 countries, CHWs have become an essential link in health systems. Acting as intermediaries between formal healthcare structures and communities, they are often the only point of access to healthcare in rural areas, a situation that is even more critical in low-income countries such as Madagascar. In this predominantly rural country, poverty severely limits access to quality health services. At the same time, TB and HIV continue to have high and even increasing prevalence rates and disease burden, exacerbated by stigma that hinders screening and treatment.

The Ministry of Public Health estimates that less than 5% of community health workers in Madagascar are involved in TB and HIV control activities. This low level of involvement can be explained in particular by the absence of a national strategy clearly defining their role, insufficient guidelines on their training and the necessary equipment, and the lack an appropriate financial support mechanism, and other systemic obstacles.

To address this challenge, the NGO Doctors for Madagascar (DFM) and its academic partner, Charité – Universitätsmedizin Berlin, brought together 37 community health actors, including CHWs, other field workers, NGO representatives, and ministry officials for an in-person workshop in March 2025. Together, they identified and developed appropriate, feasible, and immediately applicable solutions to strengthen the involvement and impact of CHWs in the fight against TB and HIV.

Objective

This document aims to present the recommendations developed following this expert workshop and thus serves as a guide for implementing solutions adapted to the local context, with a view to strengthening the involvement and impact of community health workers in the fight against TB and HIV in Madagascar.

Workshop methodology

A three-day in-person workshop, based on the design thinking approach, was organized to develop an operational strategy aimed at strengthening the involvement and impact of CHWs in the fight against HIV and TB. The event brought together CHWs, supervisors, and health workers from the regions Analamanga, Itasy, and Atsimo-Andrefana, as well as representatives from NGOs (DFM, PSI, SALFA, ASOS, SAF/FJKM), EMAD and EMAR teams from Itasy and Atsimo-Andrefana, and representatives from the DPS, DSSB, DLMT, PNLIST, and PNLT.

The method aimed to stimulate creativity, encourage engagement, and ensure participation from everyone, regardless of level or sector. The program alternated between presentations, group work, individual exercises, and plenary discussions. The groups, which were formed in advance to ensure balanced representation, encouraged free expression.

Special attention was given to CHWs from diverse backgrounds, who were invited to share their experiences, working conditions, and constraints. Their advocacy was supported by evidence from a mixed-methods study conducted by DFM and Charité - Universitätsmedizin Berlin, providing a clear picture of their socioeconomic characteristics, motivations, obstacles, and challenges.

The workshop was designed around the following three phases of design thinking:

1. ***Empathy and problem definition***: Participants put themselves in the shoes of a CHW to list daily tasks, tools, successes, and obstacles.
2. ***Ideation***: A wide range of ideas emerged thanks to everyone's input and feedback.
3. ***Elaboration***: The selected proposals were transformed into specific, achievable actions tailored to the context.

By placing CHWs at the heart of the process, the workshop produced concrete, tailored, and applicable solutions rather than mere theoretical recommendations.

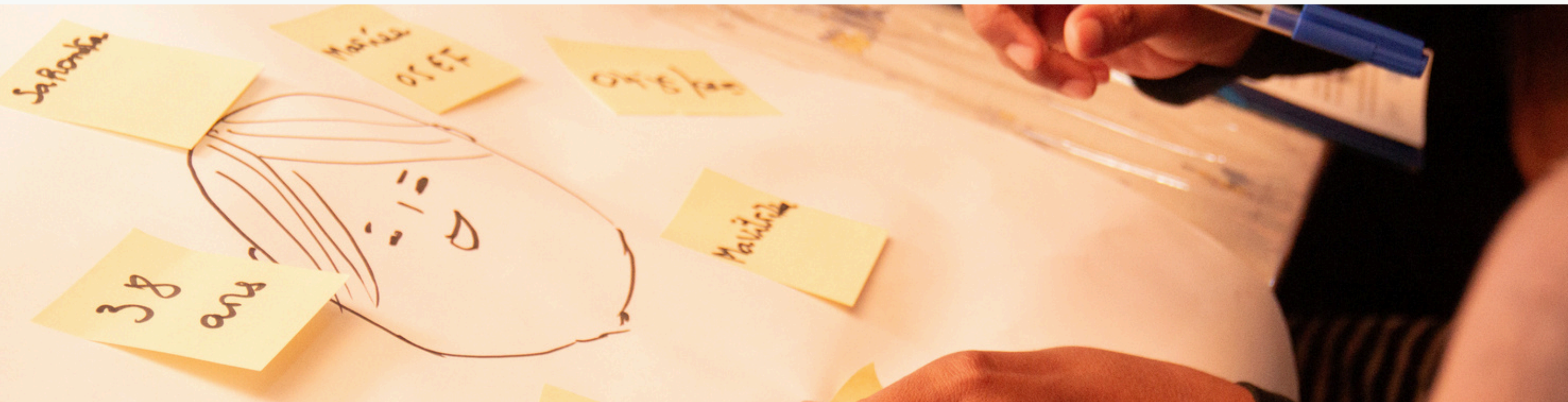

Seven levers

The workshop identified seven key levers for strengthening the response to TB and HIV by organizations working with CHWs in Madagascar. These are summarized in the acronym PROFESS, with each letter corresponding to a theme detailed in the table below:

	Levers & objectives	***Recommended items***
**P**	**Financial Protection**<br>*Ensuring fair and sustainable compensation for CHWs*	• Establishment of transparent, regular, and proportional compensation<br>• Support for health coverage<br>• Support for income generation
**R**	**Recognition**<br>*Promoting the role of CHWs*	• Establishment of an official recognition system by local authorities<br>• Community involvement in promoting CHWs<br>• Promotion of cohesion among CSB and CHW staff
**O**	**Coordination**<br>Harmonizing interventions around CHWs	• Strengthening multisectoral coordination<br>• Utilizing the quarterly meetings of the CCDS<br>• Presenting and validating community health workers' action plans during these meetings<br>• Clarifying the roles and responsibilities of CHWs.<br>• Aligning all activities with national guidelines<br>• Establishing a simple, shared reporting system for CHWs
**F**	**Formation (Training)**<br>*Strengthening the skills of CHWs*	• Establishment of a schedule for regular and ongoing training<br>• Ensure access to training for all CHWs, including those in remote areas<br>• Adaptation of training content to relevant topics (TB, HIV, communication, etc.)<br>• Evaluation and monitoring of the impact of training

**E**	**Equipment**<br>*Ensuring the necessary equipment and materials for CHWs*	• Provision of simple, functional tools adapted to the local context<br>• Regular and equitable distribution of necessary equipment and materials<br>• Maintenance and replacement of worn equipment
**S**	**Supervision**<br>*Constructively supporting the performance of CHWs*	• Implementation of a supportive and structured supervision system<br>• Plan regular field visits by supervisors<br>• Train supervisors in constructive and personalized feedback<br>• Strengthen the relationship between supervisors and CHWs
**S**	**Security**<br>*Protecting CHWs in their role*	• Guaranteed access to personal protective equipment<br>• Enhanced physical security for CHW during travel, including protection against environmental and social risks<br>• Support in the event of an incident or threat

OPERATIONAL RECOMMENDATIONS

P	Financial Protection

Challenges	Objectives
• Compensation too low and paid irregularly<br>• Personal expenses not reimbursed	1. Adjust allowances to reflect actual workload<br>2. Ensure regular and transparent payment<br>3. Provide basic social security coverage for CHWs and their families<br>4. Strengthen income-generating activities (IGAs)

Actions

P1: Revision of the compensation system

● Identify all CHWs, including their contact details, mapping of activities, actual involvement, training and equipment received, and collaborating NGOs.

● Define a compensation scale :

○ **Fixed allowance** with clearly defined expected activities (e.g., 50,000 Ary /month)

○ **Reimbursement of transportation costs** if not included in the fixed allowance (e.g., 100 km × 500 Ary/km), and other personal expenses (e.g., purchase of PPE, travel, patient referrals, communication expenses).

○ **Performance bonus** if not covered by the fixed allowance (e.g., 2,000 to 10,000 Ary per detected case)

○ **Training participation allowances** aligned with those specified in the MEO guide: Accommodation outside the fokontany at 10,000 Ariary, daily allowance of 15,000 Ariary.

● Develop and distribute technical data sheets for each type of compensation, clearly detailing the criteria, terms, frequency, and amounts.

● Consult with the DSSB to validate the proposed grids.

P2: Payment security

● Choose the payment method (Mobile Money, cash) with the relevant CHWs, taking into account network accessibility constraints.

● Track and justify transactions using a simple and transparent digital tool or register.

● Train CHWs and supervisors in the use of the tool.

● Establish and publish a monthly payment schedule that ensures regular payments by setting specific dates each month, designating individuals responsible for the process, and including a clear monitoring and evaluation strategy.

P3: Health coverage for CHWs and their families

● **Assess health coverage needs** for the concerned CHWs, including common illnesses, expenses incurred, vulnerabilities.

● Establish a **social protection** or **health insurance system** (e.g., contributions, mutual insurance companies, subsidies, equity funds).

● Negotiate opportunities to join a **mutual insurance company** or **existing schemes.**

P	Financial Protection
P4: Support for IGAs<br>● Support the creation of **CHWs associations** by CSB (e.g., support for structuring, member elections, statutes, administrative registration).<br>● Training associations in **micro-project management**.<br>● Provide **kits for IGAs** according to the type of economic activity of the family or CHW association.<br>***P5 : Free education***<br>● A call for an interagency agreement to waive school registration fees for children of CHWs.	

R	Recognition
**Challenges**<br>• Lack of a formal recognition framework<br>• Informal recognition is not very motivating	**Objectives**<br>1. Establish official recognition by the government<br>2. Publicly celebrate deserving CHWs<br>3. Strengthen cohesion between CHWs and healthcare staff

Actions

R1: National distinction system

● Develop a **formal recognition framework** (e.g., certificate or honorary medal) awarded by the state or local health authorities.

● Define **selection criteria** while taking into account the indicators in the CHW motivation manual.

● Involve **local and health authorities** and the **CCDS** in the selection and award process.

● Organize **public ceremonies** to award medals or certificates on the occasion of health events (World Health Day, etc.) to add weight to the recognition given.

R2: Simplification of administrative procedures

● Facilitate access to administrative documents at local administrative centers for CHWs and their families (e.g., copies of birth certificates, residence certificates).

R3: Promotion of cohesion

● Cohesion between CHW and healthcare staff through team building activities or shared meals.

O	CoOrdination

Challenges	Objectives
• Poorly defined roles for stakeholders<br>• Fragmented coordination<br>• Complex reporting tools	1. Update guidelines<br>2. Simplify the reporting system<br>3. Promote multisectoral coordination<br>4. Share activity plans among all stakeholders

Actions

O1: Principle "One approach - one budget - one plan"

Use the quarterly meetings of the CCDS as the **primary mechanism for multisectoral coordination**, involving community health workers, health workers, local authorities, and TFPs.

O2: Action plan

During the monthly meeting, CSB leaders assist the CHWs in developing their action plans.

O3: Digital reporting

Establish an **electronic reporting system** adapted to the local context in collaboration with health facilities.

F	Formation (Training)
**Challenges**<br>• Content not well suited to the context<br>• Lack of local trainers	**Objectives**<br>1. Adapt modules to regional needs<br>2. Create a pool of trainers at the CSB level<br>3. Integrate TB/HIV into a single module<br>4. Ensure quarterly retraining

Actions

F1: Needs assessment

● Conduct a **detailed analysis** of the **training needs of CHWs**, region by region.

● **Adapt training modules** accordingly based on identified needs.

F2: Integrated TB/HIV module

● Design a **single TB/HIV module** in collaboration with the PNLIST and the PNLT.

● Organize in-person, interactive sessions focused on sharing experiences and networking.

● Schedule a follow-up session one month after the training and a refresher course every six months.

● Plan training sessions by health focus (during the dry season).

● Conduct formative supervision during the monthly meeting and during on-site supervision sessions.

F3: Training of trainers

● Prioritize **training CDT managers** and **CSB facility heads** in both basic content and specific topics so that they can transfer this knowledge to CHWs.

F4: Impact monitoring

● Hold regular meetings with CDT managers or CSB facility heads to **assess the impact of training** on CHW performance.

● Conduct practical **on-site supervision** to consolidate knowledge and strengthen skills in a sustainable manner.

E	Equipment

Challenges	Objectives
• IInsufficient or poorly distributed resources<br>• Neglected maintenance	1. Establish a detailed register of needs<br>2. Ensure fair and sustained resource allocation<br>3. Implement a participatory maintenance plan

Actions

E1: Inventory and prioritization

● Conduct a **participatory inventory of existing equipment and needs**, region by region, with the contribution of CHWs, CSB facility heads, EMADs, and EMARs.
● Classify and **prioritize equipment** based on the daily **usefulness** to **CHWs** and the local context.
● Negotiate **coverage of all CHWs** through the synthesis of needs with TFPs via the PNLT and PNLIST.

E2: Logistics and traceability

● Work with TFPs, PNLIST, PNLT, EMAR, and EMAD to define a **list of essential equipment** and a **delivery schedule**.
● Centralize storage at **CSB level** and deploy an **electronic register to track receipt and distribution.**
● Schedule a **semi-annual inventory** and **annual assessment** of the impact of equipment on CHW performance, in collaboration with CSBs.

E3: Community maintenance

● Raise awareness and involve the community (repair work, periodic cleaning).
● Establish **local equipment monitoring committees**.
● Mobilize **financial support** for **maintenance and replacement parts**.

S	Supervision

Challenges	Objectives
• Rare visits perceived as "punitive"<br>• Lack of resources and tools	1. Promote formative and supportive supervision<br>2. Standardize and digitize tools<br>3. Systematically include TB/HIV

Actions

Su1: Culture of kindness

● Establish formative **supervision focused** on **support rather than control**.

● Before each training session, lead short modules explaining to CHWs the actual objectives and concrete benefits of supervision.

Su2: Field visits

● Schedule **quarterly supervisory visits** in the field, taking advantage of the advanced strategies implemented by CSB facility heads to **pool travel arrangements**.

● **Systematically integrate TB** and **HIV components** into existing supervision frameworks.

Su3: Standardized tools

● **Leverage existing tools**: including, among others, the routine monitoring checklist for CHWs.

● Develop simplified reporting tools - and, where possible, digitized ones - **to supplement official templates**, ensuring they are compatible with local connectivity.

S	Security

Challenges	Objectives
• Long journeys, sometimes at night<br>• Limited protective equipment	1. Reduce risks associated with travel<br>2. Implement working in pairs<br>3. Mobilize local authorities to protect CHWs

Actions

Se1: Reduce travel

● Prioritize recruiting **CHWs who live in the areas of operation**, especially in the most remote locations, in order to limit the travel distances.

Se2: Organization and PPE

● Establish a **mandatory buddy system** for all long trips, overseen by the CSB facility head.

● Work with the CHWs to develop a **travel schedule** that is communicated in advance to community leaders.

● Provide a **complete protection and visibility kit** (fluorescent vest, headlamp).

Se3: Support from the authorities

● Integrate **CHW security into the "Dina Be" community charter**, in collaboration with mayors, fokontany chiefs, and religious/traditional leaders.

● Set up an emergency hotline, jointly managed by the Ministry of Public Health and TFPs.

Acting together

The **PROFESS** guide provides a clear roadmap; its success depends on collective and coordinated commitment. It is not intended to create further fragmentation or to restrict its use solely to strengthening CHW engagement in the response to TB and HIV: its principles are fully transferable to other areas of health (vaccination, maternal and child health, etc.) as well as to other community profiles requiring structured strengthening, such as peer educators.

We call on all actors involved in community health in Madagascar to:

- **Establish**: Officially adopt PROFESS as the national standard and integrate it into TB/HIV policies and other priority health programs.
- **Finance**: Secure resources for compensation, continuing education, essential equipment, and digital supervision.
- **Implement**: Translate each lever into simple quarterly actions (one-time payments, unified TB/HIV modules, simplified reporting, available PPE, etc.) and adapt them for other community interventions.
- **Monitor and adjust**: Use common indicators, such as CHW coverage, diagnostic delays, CHW satisfaction, and revise strategies based on representative field data, promoting inter-program harmonization.
- **Promote**: Publicly recognize community volunteers, ensure their safety, and encourage community participation in equipment maintenance.

By drawing inspiration from PROFESS and working closely together, stakeholders will be able to build a truly comprehensive community healthcare system that benefits all Malagasy populations.

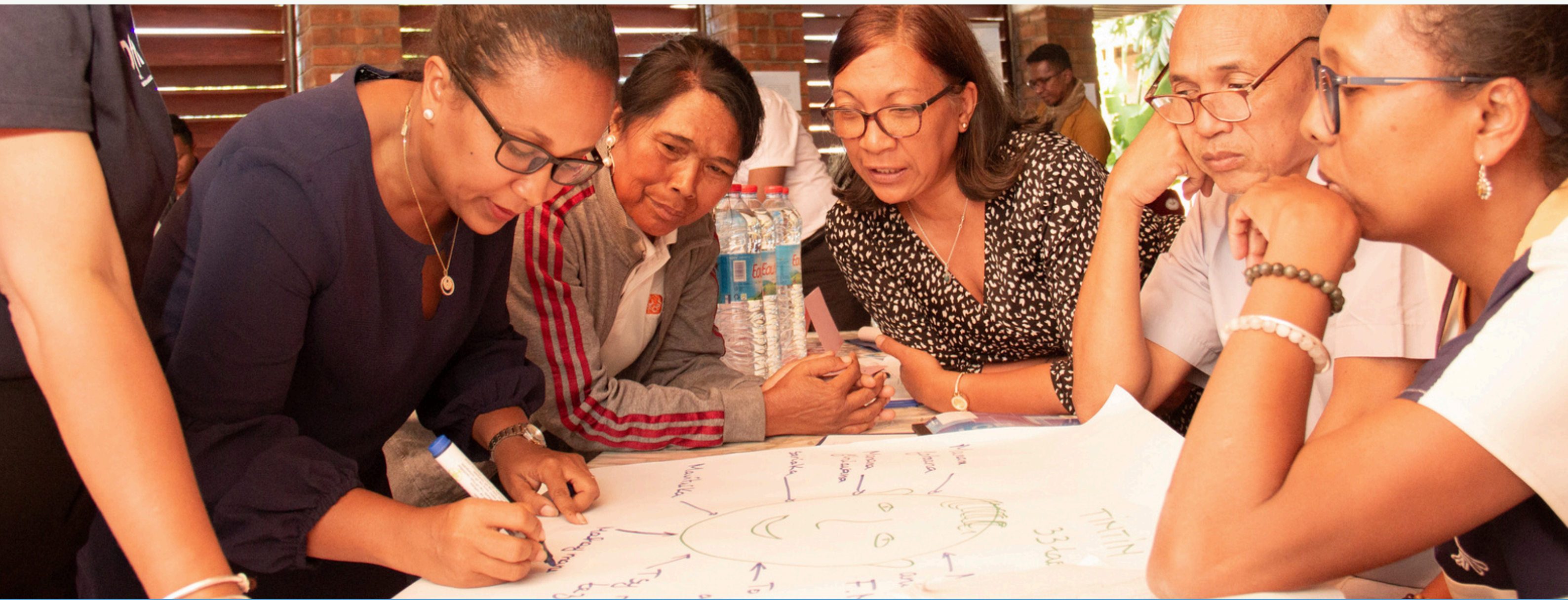

PROFESS Guide

Strengthening community health workers in the response to tuberculosis and HIV in Madagascar: seven levers for action

Recommendations from a design thinking workshop

Madagascar, March 2025

Main Authors: Onja Gabrielle **Ravololohanitra**, Michel Harison **Tiaray**, Norosoa Assunta **Rakotoherimora**, Haingotiana **Razafimanantsoa**, Larissa **Hecht**, Nadine **Muller**
