# Supplementary material for: Co-designing PROFESS: a community health worker support framework to strengthen engagement in TB and HIV care in Madagascar
Source: Front Health Serv. 2026 Sep 18;6:1910840. doi: 10.3389/frhs.2026.1910840 (PMC13631075; doi:10.3389/frhs.2026.1910840)
Supplement: Supplementary file 3 [file Datasheet3.pdf]

SUPPLEMENTARY MATERIAL 2

Manuscript: Co-designing PROFESS: a community health worker support framework to strengthen engagement in TB and HIV care in Madagascar

Ravololohanitra et al.

Individual Interview Guide

Guide for Interviews with Stakeholders Involved in Community-Based Tuberculosis Care

1. Introduction

- Introduction of the interviewer and the study.
- Information on good scientific practices, including confidentiality.
- Obtaining informed consent.
- Collection of demographic information: age, sex, place of residence.

2. General Context

Questions:

-Could you tell us more about your role in relation to Community Health Workers (CHWs)?

Probe:

- Your working relationship with CHWs.
- How long you have been working with CHWs.
- Whether this relationship is permanent or temporary.

3. Opportunities and Challenges faced by Stakeholders in Their Collaboration with CHWs

Questions:

-From your perspective, what are the main benefits and strengths of integrating CHWs into the health system, both in general and in the context of your own work?

Probe:

- Improved access to healthcare.

- Proximity.
- Economic benefits.
- Better organization of healthcare activities.

-What challenges or barriers do you encounter when working with CHWs, both in the broader context of healthcare and specifically in relation to your tuberculosis control activities?

Probe:

- Limitations in CHW skills or competencies.
- Supervision.
- Motivation.
- Materials and equipment.
- Organization.

-From your point of view, among the challenges you have mentioned, which do you consider to be the most important when working with CHWs, both in general healthcare and specifically in tuberculosis-related activities?

4. Stakeholders' Needs

Perceived needs

Identification of needs

Questions:

-What specific forms of support would help improve your ability to collaborate effectively with CHWs?

Probe:

- Materials.
- Equipment.
- Technical expertise.
- Financial or other resources.
- Policies.
- Organizational structures.

-In your opinion, do the conditions required to collaborate with CHWs vary depending on the health topic or disease area? If so, could you explain why?

Probe:

- Specific needs related to infectious diseases such as tuberculosis and HIV.

5. Interaction with CHWs and Supervision

Triggers for communication and interaction

Assessment of interaction and supervision

Questions:
-Could you tell me more about how you communicate with CHWs?

Probe:

- Communication channels used.
- Frequency of communication.
- Types of topics discussed.

-In your opinion, why is it necessary to supervise CHWs in their roles?

Probe:

- Quality of services.
- Performance monitoring.
- Maintaining motivation.

-Could you describe what you consider to be an ideal supervision structure for CHWs?

-Who would be the ideal stakeholder or person to supervise CHWs?

Probe:

- The stakeholders involved .

6. Motivation and Collaboration

Questions:
-In your opinion, how do CHWs determine their priorities across the different areas of their activities?

Probe:

- Incentives associated with specific activities or health topics.
- Collaboration with NGOs.
- Effectiveness of activities.
- Available support.

-During your collaboration with CHWs, have you encountered any challenges related to their motivation, such as attrition or other issues?

Probe:XXX

-From your perspective, what type of motivation system should be explored to ensure the sustainability of CHW activities?

Probe:XXX

-Based on your personal experience, how do you perceive the support provided by national policy for voluntary CHW work?

Probe:XXX

-Do you have any particular experience in implementing a motivation system for CHWs? If so, could you briefly describe the successes you achieved, as well as the facilitators and barriers you encountered?

Probe:

- On what basis?
- What form ?
- How frequently was it provided?
- Recognition.
- Equipment…

7. Recommendations

-If you were responsible for reviewing the policy governing the roles of CHWs in tuberculosis care and for providing key recommendations to improve their effectiveness and sustainability, what specific aspects would you suggest changing?

Probe:

- For each recommendation, explore: How should this change be implemented?
- Why do you think this change is necessary?

8. Conclusion

- Ask the participant whether there are any other questions they would like to discuss.
- Explain the next steps in the research process, including feedback and dissemination plans.
- Provide the contact details of the relevant person and invite the participant to contact them if they have any further questions.
- Thank the participant for their time and participation.

Emoticon

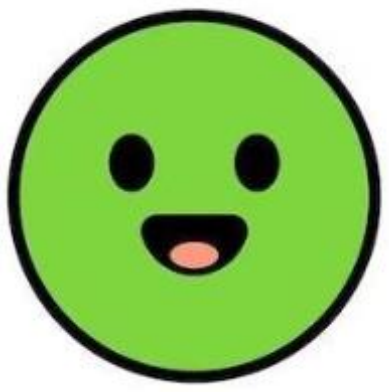

: completely agree

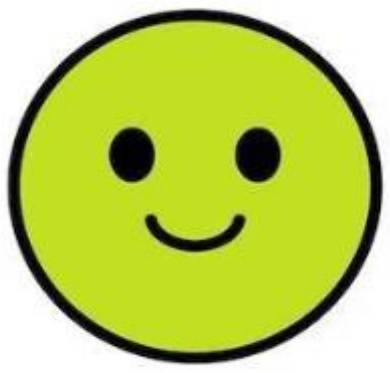

: Agree

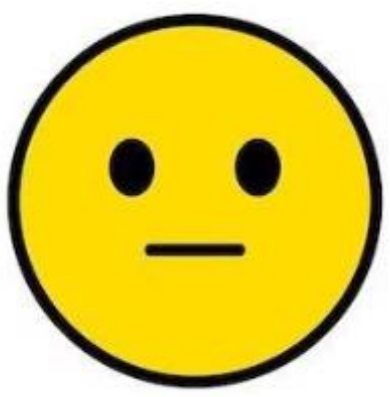

: Neither agree nor disagree

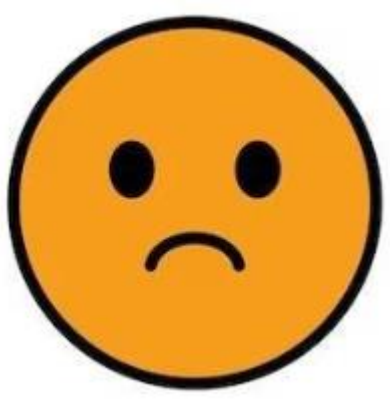

: Disagree

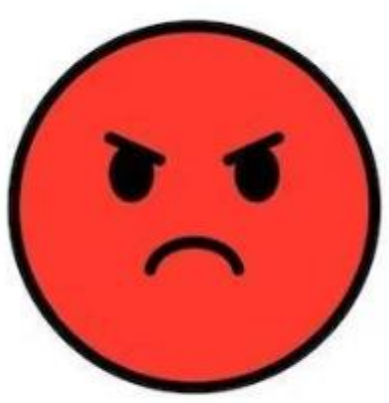

: Completely disagree
