# Supplementary material for: Co-designing PROFESS: a community health worker support framework to strengthen engagement in TB and HIV care in Madagascar
Source: Front Health Serv. 2026 Sep 18;6:1910840. doi: 10.3389/frhs.2026.1910840 (PMC13631075; doi:10.3389/frhs.2026.1910840)
Supplement: Supplementary file 4 [file Datasheet4.pdf]

SUPPLEMENTARY MATERIAL 2

Manuscript: Co-designing PROFESS: a community health worker support framework to strengthen engagement in TB and HIV care in Madagascar

Ravololohanitra et al.

- *English translation: pages 1-5*
- *French translation: pages 6-8*

Focus Group Interview Guide / Community Health Worker (CHW)

Main Theme

1. Introduction

Questions:

- Introduction of the interviewer and the study
- Information on scientific best practices, including confidentiality
- Obtaining informed consent
- Collection of sociodemographic data (affiliated health organization, age, sex, region).

2. Recruitment of CHWs

- How does one become a CHW?
- What are the selection criteria?
- Who are the decision-makers?

- Questions:

2.1. From your perspective, what are the characteristics of a good CHW?

Probing:

- Typical profile, required attitudes and skills

2.2. How does the selection process take place? What are the criteria?

Probing:

- Is the selection method the same or different depending on the location or other factors? Who defines the method? How do you assess the criteria?

2.3. Who is the most influential person in the selection of CHWs?

Probing:

- his opinion of the decision-maker

2.4. What are your recommendations to improve the selection process to better meet community needs?

Probing:

- Recommendations on the selection process, criteria, and decision-maker

3. Work Environment

- Challenges Encountered

- Amenities
- Commitments

Questions:

3.1. From your perspective as a CHW, how would you describe your general work environment?
Probing:

- Atmosphere of work (good or difficult), workload, workplace safety, risks, relationships with supervisors and among peers.

3.2. In your opinion, what are the most important aspects? Why?

Probing:

- intrinsic and personal motivations such as recognition and gratitude, or material motivations.

Perception and Motivation of CHWs

How do community health workers view their role within the community and the health system?

Questions:

4.1. How do you perceive your role as a CHW within the health system?
Probing: To what extent do you feel indispensable? If CHWs were absent, what could happen?

4.2. How do you perceive your role as a CHW within your community?

Probing: In relation to those with difficult access to care, those who are distant, more vulnerable, or stigmatized.

4.3. What was your initial motivation to become a CHW?

Ask for some stories about the future of CHWs?

4.4. How is your current motivation different from your initial motivation? What has changed?

Probing: material and non-material motivations.

4.5. What motivates you to work as a CHW, and what is the most motivating factor?

4.6. What demotivates you in your work as a CHW, and what is the most discouraging factor?

5. Volunteering and Financial Compensation

- Incentives and Income
- The Importance of Volunteering
- Factors That Increase the Sustainability of Initiatives

Questions:

5.1.What do you earn as a CHW?

Probing: remuneration, allowances, equipment.

5.2. What constraints do you face in voluntarily carrying out your tasks?

6. Recommendations to Better Support CHWs

- Practical Tips
- Support in Various Areas

Questions:

6.1. Imagine you had a magical power to make one wish that could improve the situation of CHWs. What would your wish be?

6.2. In your opinion, what could improve the situation of CHWs in effectively accomplishing their tasks? You may reflect on different areas of life: What could you personally do? What could the community do? What could politicians and other decision-makers do?

Probing: equipment, incentives, supervision, training, performance feedback, recognition, gratitude.

7. Engagement in Communicable Diseases (TB & HIV/AIDS)

Questions:

7.1. Are there factors that prevent or discourage CHWs from engaging in the fight against these diseases? (e.g., lack of NGOs, lack of training, stigma, risk of infection, access to affected populations)

7.2. What specific recommendations would you make to increase CHWs' engagement in these areas? Other questions?

8. Conclusion

Questions:

8.1. . Would you like to add anything?

8.2. Any other questions?

- Information on next steps (data collection, data analysis, dissemination)
- Reminder of contact details and possibility to reach out if questions arise later.

Thanks and acknowledgments

Guide d’entretien - Groupe de discussion focalisé

Guide d’entretien pour Focus group discussion / Agent communautaire

Thème principal

1. Introduction

Questions :

- Présentation de l'enquêteur et de l'étude
- Informations sur les bonnes pratiques scientifiques, y compris la confidentialité
- Obtention du consentement éclairé
- Collecte de données sociodémographiques (Organisme sanitaire d’affiliation, âge, sexe, région).

2. Recrutement des ASCs

- Comment devenir un ASC ?
- Critères de sélection
- Décideurs

Questions :

2.1. Selon votre point de vue, quelles sont les caractéristiques d'un bon ASC ?

Sonder : le profil type, attitude et compétences requise pour devenir ASC

2.2. Comment se déroule le processus de sélection pour devenir ASC ? Quels sont les critères de sélection ?

Sonder :

Mode de sélection : le même ou différent selon les lieux ou autres causes. Qui définit le mode de sélection ? Comment appréciez-vous les critères de sélection ?

2.3. Qui est la personne la plus influente dans la sélection des ASC ?

Sonder : son avis sur le décideur

2.4. Quelles sont vos recommandations sur la manière d'améliorer la sélection des ASC afin de mieux couvrir les besoins de la communauté ?

Sonder : recommandations sur le mode de sélection, les critères et le décideur

3. Environnement de travail

- Les difficultés rencontrées
- Les commodités
- Les engagements

Questions :

3.1. Dans vos perspectives en tant qu'ASC, comment décrivez-vous votre environnement de travail en général ?

Sonder : l'atmosphère de travail : bon ou difficile, les charges de travail, la sécurité au travail, les risques, les relations avec les supérieurs, les superviseurs et entre eux-même.

3.2. Selon votre opinion, quels sont les aspects les plus importants ? Pourquoi ?

Sonder : les intrinsèques et motivations personnelles comme reconnaissance et gratitude ou motivations matérielles.

4. Perception et motivation des ASC

- Comment les ASC perçoivent-ils leur rôle au sein de la communauté et du secteur de santé ?

Questions :

4.1. Comment percevez-vous votre rôle d'ASC au sein du système de santé ?
Sonder : À quel point se sentent-ils indispensables, si les ASC manquent, que pourrait-il arriver?

4.2. Comment percevez-vous votre rôle d'ASC au sein de votre communauté ?
Sonder : par rapport à ceux qui ont des difficiles accès aux soins, ceux qui sont loins, plus vulnérables et stigmatisés

4.3. Quelle a été votre motivation initiale pour devenir ASC ?
Demander quelques histoires sur le devenir des ASC?

4.4. En quoi votre motivation actuelle est-elle différente de votre motivation initiale ? Que s'est-il passé ?
Sonder : les motivations matérielles, non matérielles

4.5. Qu'est-ce qui vous motive à travailler en tant que ASC et quel est le facteur le plus motivant ?

4.6. Qu'est-ce qui vous démotive en travaillant en tant que ASC et quel est le facteur plus démotivant ?

5. Volontariat et récompense financière

- Incitations et revenus
- Importance du volontariat
- Facteurs augmentant la durabilité des actions

Questions :

5.1. Que gagnez-vous en tant que ASC ?
Sondez : rémunérations, indemnités, équipements

5.2. Quelles sont les contraintes auxquelles vous êtes confronté(e) dans l'accomplissement volontaire des tâches ?

6. Recommandations pour mieux soutenir les ASC

- Suggestions pratiques
- Support dans différents domaines

Questions :

6.1. Imaginez que vous disposiez d'un pouvoir magique vous permettant de réaliser un vœu qui pourrait améliorer la situation des ASC. Quel serait votre souhait ?

6.2.

6.3. Selon vous, qu'est-ce qui pourrait améliorer la situation des ASC dans l'accomplissement efficace de leurs tâches ? Vous pouvez réfléchir à différents domaines de la vie : Que pourriez-vous faire personnellement ? Que pourrait faire la communauté ? Que pourraient faire les hommes politiques et les autres décideurs ?
Sonder : Équipement, incitation, supervision, formation, retour sur les performances, reconnaissance, gratitude.

7. Engagement pour les maladies transmissibles (TB & VIH/SIDA)

7.1. Existent-ils des facteurs qui empêchent ou laissent hésiter les ASC à s'engager dans la lutte contre ces maladies ? (p.ex. manque d'ONG, manque de formation, stigma, risque d'infection, accès aux populations affectées)

7.2. Quelles sont les recommandations spécifiques pour accroître l'engagement des ASC dans ces domaines ? Autres questions ?

8. Conclusion

Questions :

8.1. Vous souhaitez ajouter quelque chose ?

8.2. D'autres questions ?

- Informations sur les prochaines étapes (collecte des données, analyse des données, diffusion)
- Rappel des coordonnées et de la possibilité de prendre contact si des questions se posent ultérieurement.
- Remerciements
