# Supplementary material for: Co-designing PROFESS: a community health worker support framework to strengthen engagement in TB and HIV care in Madagascar
Source: Front Health Serv. 2026 Sep 18;6:1910840. doi: 10.3389/frhs.2026.1910840 (PMC13631075; doi:10.3389/frhs.2026.1910840)
Supplement: Supplementary file 5 [file Datasheet5.pdf]

SUPPLEMENTARY MATERIAL 1

Manuscript: Co-designing PROFESS: a community health worker support framework to strengthen engagement in TB and HIV care in Madagascar

Ravololohanitra et al.

- *English translation: pages 1-22*
- *French translation: pages 23-43*
- *Malagasy original version: pages 44-61*

Questionnaire on the characteristics of Community Health Workers (CHWs) in Madagascar

A. Consent and eligibility verification

A.1. You are aware that participation in this study is voluntary.

o The respondent agrees with the statements above and wishes to participate in the survey o The respondent does not agree to participate in the survey

A.1'. o Male or o Female (by observation)

A.2. Are you at least 18 years old?

o Yes

o No

A.3. Have you been working as a CHW for more than one year?

o Yes

o No

B. Work environment

In the following section, I will ask you a few questions about your work environment as a CHW.

B.1. How did you become a CHW?
(Multiple-choice question; several answers possible – instructions from the training manual) ☐ Recommended by another CHW

☐ Selected by a Fokontany leader

☐ Elected during a community meeting (by the community)

☐ Selected by the mayor or the social development committee (commune

level) ☐ Selected by a non-governmental organization (NGO)

☐ Selected by a religious leader

☐ Selected by a health worker from a health facility

☐ Other

☐ Does not know

☐ Refuses to answer

B.1.1. If "Other" in B.1, please specify: ________________________

o Does not know
o Refuses to answer

B.2. As a CHW, do you work in a field other than health?

o Yes

o No

o Does not know

o Refuses to answer

B.2.1. If "Yes" in B.2, in which field?

o Does not know

o Refuses to answer

B.2'. As a CHW, was an official presentation made to the community stating that you are officially part of the CHW workforce?

o Yes

o No

o Does not know

o Refuses to answer

B.3. After officially becoming a CHW, how many years of experience do you have as a CHW? (Use whole numbers only. Round to the nearest whole number.)
__________ year(s)

o Does not know

o Refuses to answer

B.4. In how many Fokontany do you work as a CHW?

o Does not know

o Refuses to answer

B.5. As a CHW, what is your usual work frequency?

o Every day of the week

o One or more fixed days per week

o Variable frequency depending on another job

o Variable frequency depending on a factor other than work

o Does not know

o Refuses to answer

B.5.1. If “Variable frequency depending on a factor other than work,” please

specify: o Does not know

o Refuses to answer

B.6. On average, how many hours per week do you devote to your role as a CHW? (Use whole numbers only. Enter 0 for less than one hour per week.)
__________ hours per week

o Does not know

o Refuses to answer

B.7. How do you access the communities you are responsible for? (Multiple-choice)

☐ On foot

☐ By bicycle

☐ By public transport

☐ By canoe

☐ Other means of transport

☐ Does not know

☐ Refuses to answer

B.7.1. If “Other means of transport,” please

specify:____________________ o Does not know

o Refuses to answer

B.8. What equipment do you have access to in order to carry out your work as a CHW? (Multiple-choice)

☐ Health education tools (e.g. flipcharts, books)

☐ Work guide or guidelines

☐ First aid or emergency kit

☐ Backpack

☐ Monitoring notebook

☐ Uniform (e.g. vest, cap)

☐ Bicycle

☐ Mobile phone

☐ Megaphone

☐ Other

☐ No equipment

☐ Does not know

☐ Refuses to answer

B.8.1. If "Other," please specify:___________________________

o Does not know

o Refuses to answer

B.9. Which techniques have you used as a CHW over the past two years? (Multiple-choice)

☐ One-on-one meetings (e.g. home visits)

☐ Group discussions

☐ Counseling

☐ Mass awareness activities (megaphone, "tam-tam")

☐ Other

☐ Does not know

☐ Refuses to answer

B.9.1. If "Other," please specify:_____________________________

o Does not know

o Refuses to answer

C. Services provided

In the following section, I will ask you about the services you may have provided over the past two years.

Tuberculosis

C.1. As a CHW, have you participated in tuberculosis (TB) prevention or control over the past two years?

o Yes

o No

o Does not know

o Refuses to answer

C.1.1. If “Yes,” which TB-related services did you provide over the past two years? (Multiple-choice)

☐ Awareness-raising on TB prevention measures

☐ Awareness-raising on TB transmission

☐ Awareness-raising on TB warning signs

☐ Informing the community about free TB testing and medication

☐ Identifying and referring presumptive TB cases for care
☐ Identifying and referring TB contacts for care

☐ Fighting stigma or discrimination against TB patients

☐ Promoting BCG vaccination for children under 45 days

☐ Supporting directly observed TB treatment

☐ Acting as a representative of TB diagnosis and treatment centers at community

level ☐ Communicating information to TB diagnosis and treatment centers

☐ Tracing and following up patients lost to follow-up

☐ Encouraging TB patients to undergo HIV testing

☐ Other

☐ Does not know

☐ Refuses to answer

C.1.1.1. If “Other,” please

specify:____ o Does not know

o Refuses to answer

HIV/AIDS

C.2. As a CHW, have you participated in HIV/AIDS prevention or care over the past two

years? o Yes

o No

o Does not know

o Refuses to answer

C.2.1. If "Yes," which HIV/AIDS-related services did you provide over the past two years? (Multiple-choice)

☐ Awareness-raising on HIV/AIDS prevention

☐ Awareness-raising on HIV/AIDS transmission

☐ Awareness-raising on early signs of HIV/AIDS

☐ Informing the community about free HIV testing and treatment

☐ Encouraging community members to attend health centers for genital

symptoms ☐ Identifying and referring presumptive HIV cases for care

☐ Identifying and referring contacts for HIV care

☐ Fighting stigma or discrimination against people living with HIV
☐ Acting as a representative of referral centers at community level

☐ Communicating information to referral centers

☐ Encouraging TB patients to undergo HIV testing

☐ Other

☐ Does not know

☐ Refuses to answer

Malaria

C.3. As a CHW, have you participated in malaria prevention and/or control over the past two

years? o Yes

o No

o Does not know

o Refuses to answer

C.3.1. If "Yes," which malaria-related services did you provide over the past two years? (Multiple-choice)

☐ Educating pregnant women on taking at least three doses of intermittent preventive treatment (IPT)

☐ Identifying malaria symptoms

☐ Awareness-raising on mosquito control

☐ Distributing mosquito nets and educating communities on their use

☐ Performing rapid diagnostic tests and treating uncomplicated malaria

(ACTs) ☐ Referring severe malaria cases to health facilities

☐ Other

☐ Does not know

☐ Refuses to answer

C.3.1.1. If "Other" for C.3.1, please

specify:__________________________ O Does not know

O Refuses to answer

C.4. As a CHW, which other communicable diseases have you worked on over the past two years?
(Multiple-choice question)

☐ Sexually transmitted infections (STIs)

☐ COVID-19

☐ Rabies

☐ Cysticercosis, filariasis, schistosomiasis and/or helminthiasis

☐ Plague

☐ Parasitic diseases (e.g. mass drug administration for filariasis,

cysticercosis) ☐ No other communicable diseases

☐ Other(s)

☐ Does not know

☐ Refuses to answer

C.4.1. If "Other(s)" for C.4, please specify:__________________

O Does not know

O Refuses to answer

C.5. Community-based surveillance (CBS) is an active process of community participation aimed at detecting, reporting, responding to, and monitoring health events in the community.

As a CHW, have you participated in community-based surveillance over the past two

years? O Yes

O No

O Does not know

O Refuses to answer

C.5.1. If “Yes” for C.5, which community-based surveillance–related services have you provided over the past two years?
(Multiple-choice question)

☐ Identifying diseases, conditions, or other health events in the community that pose a public health risk and recording them in community registers

☐ Participating in investigations into causes of death, including among women, newborns, and animals

☐ Officially reporting a death

☐ Reporting any unexpected or unusual occurrence of disease/cases or deaths to the

CSB ☐ Involving local leaders in describing community events and outbreaks

☐ Supporting health workers during outbreak investigations and contact tracing
☐ Participating in mapping of activities and health risks

☐ Other

☐ Does not know

☐ Refuses to answer

C.5.1.1. If “Other” for C.5.1, please

specify:____________________________ O Does not know

O Refuses to answer

C.6. As a CHW, have you been involved in any of the following areas over the past two

years? (Multiple-choice question)

☐ Adolescent and youth health (10–24 years)

☐ Family planning (FP)

☐ Antenatal care / pregnancy monitoring

☐ Maternal and neonatal follow-up, including postnatal care (0–42 days)

☐ Vaccination

☐ Nutrition (e.g. growth monitoring, vitamin A and deworming

distribution) ☐ Birth registration

☐ Early childhood development

☐ Follow-up of sick children

☐ Promotion and prevention of common childhood illnesses

☐ WASH (water, sanitation, and hygiene)

☐ Management and access to health equipment

☐ Information management for monitoring and evaluation

☐ Health surveillance and control (e.g. accidents, outbreaks, poisoning in the

community) ☐ First aid

☐ Blood donation

☐ None of the above

☐ Does not know

☐ Refuses to answer

C.7. As a CHW, which non-communicable diseases have you worked on over the past two years? (Multiple-choice question)

☐ Hypertension

☐ Diabetes

☐ Cervical and/or breast cancer

☐ Chronic respiratory diseases

☐ Other

☐ Does not know

☐ Refuses to answer

C.7.1. If "Other" for C.7, please specify:________________________

O Does not know

O Refuses to answer

C.8. Are there any other services you provide as a CHW that we have not yet asked about? O Yes

O No

O Does not know

O Refuses to answer

C.8.1. If "Yes" for C.8, please specify:___________________________

O Does not know

O Refuses to answer

D. Training

In the following section, I will ask you a few questions about any training you may have received over the past two years.

D.1. Over the past two years, have you received any training lasting one day or longer? O Yes

O No

O Does not know

O Refuses to answer

D.1.1. If "Yes" for D.1, which topics were covered in the training you received over the past two years?
(Multiple-choice question)

☐ General CHW responsibilities

☐ Promotion of priority behaviours

☐ Family planning

☐ Maternal and child follow-up

☐ Vaccination

☐ Prevention of self-medication

☐ Epidemic control

☐ Prevention of communicable diseases

☐ Control of specific diseases

☐ Road traffic accidents

☐ Promotion of WASH

☐ Management of service delivery points

☐ Community system management

☐ Other

☐ Does not know

☐ Refuses to answer

D.1.1.1. If "Control of specific diseases" for D.1.1, please

specify:___________________________ O Does not know

O Refuses to answer

D.1.1.2. If "Other" for D.1.1, please

specify:________________________________ O Does not know

O Refuses to answer

D.1.1.3. For each response other than "Other," "Does not know," or "Refuses to answer": Which organisation organised and/or funded your participation in the training? (Multiple-choice question)

☐ Health centre

☐ NGO (non-governmental organisation)
☐ District hospital

☐ Ministry of Health

☐ National TB Control Programme (PNLT)

☐ Other

☐ Does not know

☐ Refuses to answer

D.1.1.3.1. If "Other" for D.1.1.3, please

specify:______________________________ O Does not know

O Refuses to answer

E. Supervision

I will now ask you a few questions about your supervision as a

CHW. **E.1.** Do you have a *formal* supervisor?

O Yes

O No

O Does not know

O Refuses to answer

E.1.1. If “Yes” for E.1, who formally supervises your work as a CHW? (Multiple-choice question)

☐ Head of CSB/CHRD

☐ Health worker based at the health centre (other than the CSB/CHRD

head) ☐ Medical inspector

☐ Fokontany leader

☐ NGO supervisor

☐ Other

☐ I have no formal supervisor

☐ Does not know

☐ Refuses to answer

E.1.1.1. If “Other” for E.1.1, please specify:________________________________

O Does not know

O Refuses to answer

E.2. Who *actually* supervises your work as a CHW?

O Head of CSB/CHRD

O Health workers based at health centres other than CSB/CHRD heads

O Medical inspector

O Community leader

O NGO staff

O Other

O I do not have a real supervisor

O Does not know

O Refuses to answer

E.2.1. If “Other” for E.2, please specify:__________________________

O Does not know

O Refuses to answer

E.3. In your opinion, which tasks should your supervisor carry out to better support you in your role as a CHW?
(Multiple-choice question)

☐ Work planning

☐ Distribution of work-related materials

☐ Providing instructions during work

☐ Offering support and advice

☐ Evaluation of CHW activities

☐ Conflict management

☐ Liaison with other departments

☐ Monitoring and control

☐ Distribution of possible incentives

☐ Other

☐ Does not know

☐Refuses to answer

E.3.1. If "Other" for E.3, please specify:____________________

O Does not know

O Refuses to answer

E.4. Regarding your role as a CHW, whom do you currently contact to communicate and report problems?
(Multiple-choice question)

☐ Head of CSB/CHRD

☐ Health workers based at health centres other than CSB/CHRD heads

☐ Medical inspector

☐ Community leader

☐ NGO staff

☐ Other

☐ I have no strong relationship

☐ Does not know

☐ Refuses to answer

E.4.1. If “Other” for E.4, please specify:______________________

Does not know

Refuses to answer

F. Motivation

I will now read statements made by other CHWs about their motivation.
Please indicate the extent to which you agree or disagree with each statement. For the following questions, use the emoticons on the last page to collect responses. Ask participants to point to the image that best reflects how they feel when they hear the statement.

F.1. “Volunteering as a CHW is very important to me.”

• Strongly agree

• Agree

• Neither agree nor disagree

• Disagree

• Strongly disagree

• Does not know

• Refuses to answer

F.2. “Volunteering as a CHW is very important for the community I serve.”

• Strongly agree
• Agree

• Neither agree nor disagree

• Disagree

• Strongly disagree

• Does not know

• Refuses to answer

F.3. “Non-monetary benefits play an important role in my motivation as a

CHW.” • Strongly agree

• Agree

• Neither agree nor disagree

• Disagree

• Strongly disagree

• Does not know

• Refuses to answer

F.4. "Recognition from my community plays a major role in my motivation as a

CHW." • Strongly agree

• Agree

• Neither agree nor disagree

• Disagree

• Strongly disagree

• Does not know

• Refuses to answer

F.5. "The money I earn as a CHW is essential to cover my household's

needs." • Strongly agree

• Agree

• Neither agree nor disagree

• Disagree

• Strongly disagree

• Does not know

• Refuses to answer

F.6. "A long-term employment contract as a CHW would motivate me to carry out my work more regularly."

• Strongly agree
• Agree

• Neither agree nor disagree

• Disagree

• Strongly disagree

• Does not know

• Refuses to answer

F.7. "Receiving a regular fixed salary would further motivate me to perform my work as a CHW." • Strongly agree

• Agree

• Neither agree nor disagree

• Disagree

• Strongly disagree

• Does not know

• Refuses to answer

F.8. "A permanent contract as a CHW would motivate me even more, *even if* it meant a decrease in my household income."

• Strongly agree

• Agree

• Neither agree nor disagree

• Disagree

• Strongly disagree

• Does not know

• Refuses to answer

F.9. "I am willing to devote more time to my role as a CHW if it allows me to earn a predictable income."

• Strongly agree

• Agree

• Neither agree nor disagree

• Disagree

• Strongly disagree

• Does not know

• Refuses to answer

G. Income

I will now ask you a few questions about potential incentives you may have received as a CHW.

G.1. Over the past two years, as a CHW, which incentives have you received from a health actor such as a CSB, an NGO, or another organisation?
(Multiple-choice question)

☐ Financial remuneration / incentive

☐ Direct assistance

☐ Transport (e.g. bicycle, transport costs)

☐ Food / nutritional support

☐ Expression of gratitude / recognition

☐ Supervision

☐ Training

☐ Meetings

☐ Health products

☐ Other

☐ None

☐ Does not know

☐ Refuses to answer

G.1.1. If “Other” for G.1, please specify:________________________

O Does not know

O Refuses to answer

G.2. Over the past two years, as a CHW, which incentives have you received from the community? (Multiple-choice question)

O Financial remuneration / incentive

O Direct assistance (e.g. community support)

O Transport (e.g. bicycle)

O Nutritional support

O Expression of gratitude / recognition

O Other

O None

O Does not know

O Refuses to answer

G.2.1. If "Other" for G.2, please

specify:_________________________________ O Does not know

O Refuses to answer

G.3. Do you have a full-time job other than your role as a

CHW? O Yes

O No

O Does not know

O Refuses to answer

G.3.1. If "Yes" for G.3, what is your occupation?

O Manager / professional

O Skilled employee

O Unskilled employee

O Student

O Other

O Does not know

O Refuses to answer

G.3.1.1. If "Other" for G.3.1, please

specify:_______________________________ O Does not know

O Refuses to answer

H. About the participant

Finally, I have a few questions about your background.

H.1. For which type of institution have you worked over the past two years? (Multiple-choice question)

☐ Public health facility

☐ Faith-based health facility

☐ NGO

☐ Other

☐ Does not know

☐ Refuses to answer

H.1.1. If "Other" for H.1, please specify:____________________

O Does not know

O Refuses to answer

H.2. How old are you?

__________ years

O Does not know

O Refuses to answer

H.4. What is your marital status?

O Single

O Living with a partner

O Married

O Officially divorced

O Separated

O Widowed

O Does not know

O Refuses to answer

H.5. How many people, including yourself, live in your household?

____

O Does not know

O Refuses to answer

H.6. What is the highest level of schooling you have attended?

O No formal education

O Primary level

O Secondary level

O Higher education (e.g. university)

O Does not know

O Refuses to answer

H.6.1. If "No formal education" or "Primary level" for H.6:
Please read the following two sentences aloud.
(Ask the participant to read the prepared sentences and assess based on your observation.)

“TAHIONAO RY ZANAHARY ITY NOSY MAMINAY ITY, HIADANA SY HO FINARITRA! HE SAMBATRA MARINA IZAHAY”

O With great difficulty

O With difficulty

O With ease
O With great ease

O Refuses to read

H.6.2. If “No formal education” or “Primary level” for H.6: Can you perform the following calculation?

“XXXXXXXX”

O Yes

O No

O Refuses to answer

H.7. What is your household’s average monthly

income? __________ Ariary

O Does not know

O Refuses to answer

H.8. On average, how much do you earn per year as a

CHW? O < 20,000 Ariary

O 20,000 – 50,000 Ariary

O 50,000 – 100,000 Ariary

O 100,000 – 200,000 Ariary

O 200,000 – 500,000 Ariary

O 500,000 – 1,000,000 Ariary

O Other

O Does not know

O Refuses to answer

H.8.1. If “Other,” please

specify:______________________ O Does not

know

O Refuses to answer

H.9. In which region do you work as a CHW?

O Analamanga

O Anosy

O Atsimo Andrefana

O Androy

O Other

O Does not know

O Refuses to answer

H.9.1. If "Other" for H.9, please specify:___________________________

O Does not know

O Refuses to answer

H.10. Do you mainly work as a CHW in a rural or urban area?

O Rural area

O Urban area

O Both

O Does not know

O Refuses to answer

H.11. Do you have access to a phone you can use?

O Yes, a personal phone

O Yes, a shared household phone, but not a personal phone

O Yes, a public phone (taxi phone)

O Other

O No

O Does not know

O Refuses to answer

H.11.1. If "Other" for H.11, please specify:________________________

O Does not know

O Refuses to answer

H.12. Have you ever heard of Mobile Money?

O Yes

O No

O Does not know

O Refuses to answer

H.13. Have you ever used a Mobile Money service?

O Yes

O No

O Does not know

O Refuses to answer

This concludes the interview. Thank you very much for your time and your responses. They are very valuable.

Questionnaire en français

A. **Consentement et vérification des critères d'éligibilité**

A.1. Vous êtes conscient du fait que la participation à l'étude est volontaire. o La personne est d'accord avec les déclarations ci-dessus et souhaite participer à l'enquête o La personne n'accepte pas de participer à l'enquête

A.1' o Homme ou o Femme (par obesrvation)

A.2. Êtes-vous âgé d'au moins 18 ans ?

- o Oui
- o Non

A.3. Travaillez-vous en tant qu'ASC depuis plus d'un an ?

- o Oui
- o Non

B. **Environnement de travail**

Dans la section suivante, j'ai quelques questions sur votre environnement de travail en tant qu'ASC.

B.1. Comment êtes-vous devenu ASC ? *(Question à choix multiple et plusieurs réponses possibles - instructions obtenues du manuel de formation)*

- Recommandé par un autre ASC
- Choisi par un chef Fokontany
- Élu lors d'une réunion communautaire (par la communauté)
- Choisi par le maire ou le comité de développement social (au niveau de la commune)
- Choisi par une organisation non-gouvernementale (ONG)
- Choisi par un chef religieux
- Choisis par un agent de santé d'un centre de santé
- *Autre*
- *Ne sait pas*
- *Refus de répondre*

B.1.1. *Si " Autre " dans B.1. :* Veuillez préciser.

- __________
- *Ne sait pas*
- *Refus de répondre*

B.2. En tant qu'ASC, travaillez-vous dans un domaine autre que la santé ?

- Oui
- Non
- *Ne sait pas*
- *Refus de répondre*

B.2.1. *Si "Oui " pour B.2. :* Dans quel domaine ?

- __________
- *Ne sait pas*
- *Refus de répondre*

B.2'. En tant qu'ASC, est-ce qu'une présentation officielle que vous faites officiellement partie des ASC a été faite auprès de la communauté?

Oui

Non

Ne sait pas

Refus de répondre

B.3. Après être officiellement devenu ASC, combien d'années d'expérience en tant qu'ASC possédez-vous ? *(Veuillez utiliser uniquement des nombres entiers. Arrondissez au nombre entier le plus proche, par exemple "1" pour au moins 1 année mais moins d'une année et demi, "2" pour plus d'une année et demi mais moins de deux années et demi)*

- __________ année(s)
- *Ne sait pas*
- *Refus de répondre*

B.4. Dans combien de Fokontany travaillez-vous en tant que ASC ?

- _____________
- *Ne sait pas*
- *Refus de répondre*

B.5. En tant qu'ASC, quelle est votre fréquence de travail habituelle ?

- Tous les jours de la semaine
- Un ou des jours fixes de la semaine
- Fréquence variable dépendant d'un autre travail
- Fréquence variable dépendant d'un facteur autre que le travail
- *Ne sait pas*
- *Refus de répondre*

B.5.1. *Si " Fréquence variable dépendant d'un facteur autre que le travail " pour B.5. :* Veuillez préciser.

- __________
- *Ne sait pas*
- *Refus de répondre*

B.6. Combien d'heures par semaine consacrez-vous à votre rôle de ASC en moyenne ? *(Veuillez utiliser uniquement des nombres entiers. Entrez 0 pour moins d'une heure par semaine.)*

- __________ heures par semaine
- *Ne sait pas*
- *Refus de répondre*

B.7. Comment accédez-vous aux communautés dont vous êtes responsable ? *(Question à choix multiple)*

- À pied
- À vélo
- En transport en commun
- En pirogue
- Autre transport
- *Ne sait pas*
- *Refus de répondre*

B.7.1. *Si " Autre transport " pour B.7. :* Veuillez préciser.

- _________
- *Ne sait pas*
- *Refus de répondre*

B.8. A quel(s) équipement(s) avez-vous accès pour effectuer votre travail en tant qu'ASC ? *(Question à choix multiple)*

- Outils de sensibilisation (par exemple, boîte à image, livres)

- Guide de travail ou lignes directives
- Trousse de premiers secours ou trousse d'urgence
- Sac à dos
- Carnet de suivi
- Uniforme (par exemple gilet, casquette)
- Vélo
- Téléphone portable
- Mégaphone
- Autre
- Aucun équipement
- *Ne sait pas*
- *Refus de répondre*

B.8.1. *Si " Autre " pour B.8. :* Veuillez préciser.

- ○ _________
- ○ *Ne sait pas*
- ○ *Refus de répondre*

B.9. Quelles techniques avez-vous utilisées en tant que ASC au cours des deux dernières années ? *(Question à choix multiple)*

- Rencontres individuelles (par exemple visites à domicile)
- Causerie de groupe
- Conseil (" Counseling ")
- Sensibilisation de masse (mégaphone, " Tam Tam ")
- Autre
- *Ne sait pas*
- *Refus de répondre*

B.9.1. *Si " Autre " pour B.9. :* Veuillez préciser.

- ○ _________
- ○ *Ne sait pas*
- ○ *Refus de répondre*

C. **Services fournis**

Dans la section suivante, j'ai quelques questions sur les services que vous avez pu fournir au cours des deux dernières années.

C.1. En tant qu'ASC, avez-vous participé à la prévention ou à la lutte contre la tuberculose (TB) au cours des deux dernières années ?

- Oui
- Non
- *Ne sait pas*
- *Refus de répondre*

C.1.1. Si “ Oui “ pour C.1.: Quels services liés à la tuberculose (TB) avez-vous fournis au cours des deux dernières années ? *(Question à choix multiple)*

- Sensibiliser sur les mesures préventives de la TB
- Sensibiliser sur les modes de transmission de la TB
- Sensibiliser sur les signes précurseurs de la TB
- Sensibiliser la communauté sur la gratuité du dépistage et des médicaments antituberculeux
- Détecter et orienter les cas présumés TB pour une prise en charge
- Détecter et orienter les cas contact pour une prise en charge
- Lutter contre la stigmatisation ou la discrimination des patients tuberculeux
- Sensibiliser à faire vacciner au BCG les enfants de moins de 45 jours ● Supporter le traitement directement observé des patients tuberculeux ● Se servir de représentant des CDT et CT au niveau de communauté
- Communiquer des informations auprès des CDT et CT de la communauté
- Faire la recherche et suivi des “ perdus de vue ”
- Sensibiliser les malades tuberculeux au dépistage du VIH/SIDA
- Autre
- *Ne sait pas*
- *Refus de répondre*

C.1.1.1. Si “ Autre ” pour C.1.1., veuillez préciser.

- _________
- *Ne sait pas*
- *Refus de répondre*

C.2. En tant qu’ASC, avez-vous participé à la prévention ou à la prise en charge du VIH/SIDA au cours des deux dernières années ?

- Oui
- Non
- *Ne sait pas*
- *Refus de répondre*

C.2.1. *Si “ Oui “ pour C.2.:* Quels services liés au VIH/SIDA avez-vous fournis au cours des deux dernières années ? *(Question à choix multiple)*

● Sensibiliser sur les mesures préventives de la VIH/SIDA
● Sensibiliser sur les modes de transmission de la VIH/SIDA
● Sensibiliser sur les signes précurseurs de la VIH/SIDA
● Sensibiliser la communauté sur la gratuité du dépistage et du traitement VIH/SIDA ● Encourager la communauté à consulter au CSB en cas de signe anormal des organes génitaux
● Détecter et orienter les cas présumés VIH/SIDA pour une prise en charge ● Détecter et orienter les cas contact pour une prise en charge VIH/SIDA ● Lutter contre la stigmatisation ou la discrimination des patients VIH/SIDA ● Se servir de représentant des centres de référence au niveau de communauté ● Communiquer des informations auprès des centres de référence de la communauté ● Sensibiliser les malades tuberculeux au dépistage VIH/SIDA
● Autre
● *Ne sait pas*
● *Refus de répondre*

C.2.1.1. *Si " Autre " pour C.1.2.1. :* Veuillez préciser.

- _________
- *Ne sait pas*
- *Refus de répondre*

C.3. En tant que ASC, avez-vous participé à la prévention et/ou au contrôle du paludisme au cours des deux dernières années ?

- Oui
- Non
- *Ne sait pas*
- *Refus de répondre*

C.3.1. *Si " Oui " pour C.3. :* Quels services liés au paludisme avez-vous fournis au cours des deux dernières années ? *(Question à choix multiple)*

● Sensibiliser les femmes enceintes à prendre au moins trois doses de Traitement Préventif Intermittent (TPI)
● Identifier les symptômes de paludisme
● Sensibiliser sur la lutte contre les moustiques
● Distribuer des moustiquaires et sensibiliser la communauté à son utilité ● Effectuer les TDR et faire le traitement en cas de palu simple : dispenser les ACT ● Référer les personnes atteintes de palu grave aux centres de santé
● Autre
● *Ne sait pas*

- *Refus de répondre*

C.3.1.1. *Si " Autre " pour C.3.1.:* Veuillez préciser.

- ○ _________
- ○ *Ne sait pas*
- ○ *Refus de répondre*

C.4. En tant qu'ASC, sur quelles autres maladies transmissibles est-ce que vous avez travaillé au cours des deux dernières années ? *(Question à choix multiple)*

- IST - infections sexuellement transmissibles
- COVID-19
- Rage
- Cysticercose, la filariose, la bilharziose et /ou l'helminthiase
- Peste
- Parasitoses, p.ex. traitements de masse contre les filarioses, cysticercose
- Aucune autre maladie transmissible
- Autre(s)
- *Ne sait pas*
- *Refus de répondre*

C.4.1. *Si " Autre(s) " pour C.4. :* Veuillez préciser.

- ○ _________
- ○ *Ne sait pas*
- ○ *Refus de répondre*

C.5. La surveillance à base communautaire (CBS) est un processus actif de participation communautaire visant à détecter, signaler, réagir et surveiller les événements de santé dans la communauté. En tant que ASC, avez-vous participé dans la surveillance à base communautaires au cours des deux dernières années ?

- ○ Oui
- ○ Non
- ○ *Ne sait pas*
- ○ *Refus de répondre*

C.5.1. Si " oui " pour C.5.: Quels services liés à la surveillance à base communautaire avez vous fournis au cours des deux dernières années ? *(Question à choix multiple)*

- Identifier les maladies, affections ou autres phénomènes de santé dans la communauté qui représentent un risque pour la santé publique et les suivre dans les registres communautaires

- Participer à l'enquête sur les causes de décès, notamment de femmes, de nouveau-nés, d'animaux
- Signaler officiellement un décès
- Toute occurrence inattendue ou inhabituelle de maladie/cas ou de décès doit être signalée au CSB
- Impliquer les dirigeants locaux dans la description des événements communautaires et des épidémies
- Soutenir les travailleurs de la santé lors des enquêtes sur les épidémies et de la recherche des contacts
- Participer à la cartographie des activités et des risques sanitaires
- Autre
- *Ne sait pas*
- *Refus de répondre*

C.5.1.1. *Si " Autre " pour C.5.1. :* Veuillez préciser.

- o __________
- o *Ne sait pas*
- o *Refus de répondre*

C.6. En tant qu'ASC, avez-vous été impliqué dans l'un des thèmes suivants au cours des deux dernières années ?

- Santé des adolescents et des jeunes (10-24 ans)
- Planification familiale " PF "
- Surveillance pendant la grossesse - période prénatale
- Suivi maternel et néonatal - périodique et post-partum ; 0-42 jours
- Vaccin
- Nutrition ; par exemple suivi de la croissance, distribution de vitamine A et de vers
- Enregistrement des naissances
- Développement de la petite enfance
- Suivi des enfants malades
- Promotion et prévention des maladies courantes chez les enfants
- " WASH " (eau, hygiène et assainissement)
- Gestion et accès aux équipements de santé
- Gestion de l'information pour le suivi et l'évaluation
- Surveillance et contrôle de la santé (par exemple accidents, épidémies, empoisonnements dans la communauté)
- Premiers secours (premiers secours et premiers secours)
- Don de sang
- Aucun des thèmes mentionnés
- *Ne sait pas*

- *Refus de répondre*

C.7. En tant qu'ASC, sur quelles maladies NON-transmissibles avez-vous travaillé au cours des deux dernières années ? *(Question à choix multiple)*

- Hypertension artérielle
- Diabète
- Cancer du col de l'utérus et/ou du sein
- Affections respiratoires chroniques
- Autre
- *Ne sait pas*
- *Refus de répondre*

C.7.1. *Si " Autre " pour C.7.:* Veuillez préciser.

- _________
- *Ne sait pas*
- *Refus de répondre*

C.8. Y a-t-il d'autres services que vous offrez en tant qu'ASC auxquels nous n'avons pas encore posé de questions ?

- Oui
- Non
- *Ne sait pas*
- *Refus de répondre*

C.8.1. *Si " Oui " pour C.8. :* Veuillez préciser.

- _________
- *Ne sait pas*
- *Refus de répondre*

D. **Formation**

Dans la section suivante, je vais vous poser quelques questions sur des formations éventuelles dont vous avez bénéficié dans les deux dernières années.

D.1. Au cours des deux dernières années, avez-vous reçu une formation d'une journée ou plus?

- Oui
- Non
- *Ne sait pas*
- *Refus de répondre*

D.1.1. *Si " Oui " pour D.1. :* Quels sujets ont été abordés dans la formation que vous avez

suivie au cours des deux dernières années ? *(Question à choix multiple)*

- Responsabilités générales du ASC
- Promouvoir les comportements prioritaires
- Planification familiale
- Suivi de la mère et de l'enfant
- Vaccin
- Lutte contre l'automédication
- Contrôle des épidémies
- Prévention des maladies transmissibles
- Lutte contre des maladies spécifiques
- Accident de la circulation de route
- Promotion de " WASH "
- Gestion des points de livraison
- Gestion du système communautaire
- Autre
- *Ne sait pas*
- *Refus de répondre*

D.1.1.1. Si " Lutte contre des maladies spécifiques " pour D.1.1. : Veuillez préciser.

- ○ __________
- ○ *Ne sait pas*
- ○ *Refus de répondre*

D.1.1.2. Si " Autre " pour D.1.1., veuillez préciser.

- ○ __________
- ○ *Ne sait pas*
- ○ *Refus de répondre*

D.1.1.3. *Pour chaque réponse autre que " Autre ", " Ne sait pas ", et " Refus de répondre " :* Quelle organisation a organisé et/ou financé votre participation à la formation ? *(Question à choix multiple)*

- Centre de santé
- ONG - organisation non-gouvernementale
- Hôpital de district
- Ministère de la Santé
- PNLT
- Autre
- *Ne sait pas*

- *Refus de répondre*

D.1.1.3.1. *Si " Autre " pour D.1.1.3. :* Veuillez préciser.

- ○ __________
- ○ *Ne sait pas*
- ○ *Refus de répondre*

E. **Supervision**

Je vais maintenant vous poser quelques questions concernant votre supervision en tant qu'ASC.

E.1. Avez-vous un superviseur « FORMEL » ?

- ○ Oui
- ○ Non
- ○ *Ne sait pas*
- ○ *Refus de répondre*

E.1.1. *Si " Oui " pour E.1. :* Qui supervise formellement votre travail en tant qu' ASC ? *(Question à choix multiple)*

- Chef CSB/CHRD
- Agent de santé basé dans les centres de santé autres que les chefs du CSB/CHRD
- Médecin inspecteur
- Chef Fokontany
- Superviseur auprès d'une ONG
- Autre
- Je n'ai aucun superviseur formel
- *Ne sait pas*
- *Refus de répondre*

E.1.1.1. *Si " Autre " pour E.1.1.:* Veuillez préciser.

- ○ _________
- ○ *Ne sait pas*
- ○ *Refus de répondre*

E.2. Qui supervise VRAIMENT votre travail en tant qu'ASC ?

- ○ Chef CSB/CHRD
- ○ Agents de santé basés dans les centres de santé autres que les chefs CSB/CHRD
- ○ Médecin Inspecteur
- ○ Chef à domicile

- Travailleurs d'ONG
- Autre
- Je n'ai pas de vrai superviseur
- *Ne sait pas*
- *Refus de répondre*

E.2.1. *Si " Autre " pour E.2. :* Veuillez préciser.

- _________
- *Ne sait pas*
- *Refus de répondre*

E.3. Selon vous, quelles tâches devrait assumer votre superviseur pour mieux vous soutenir dans votre rôle d'ASC ? *(Question à choix multiple)*

- Planification du travail
- Distribution de matériel relié au travail
- Donner des instructions pendant le travail
- Apporter un accompagnement et des conseils
- Évaluation des activités en tant que ASC
- Gérer des conflits
- Liaison avec d'autres départements
- Monitorage et contrôle
- Distribution d'incitatifs éventuels
- Autre
- *Ne sait pas*
- *Refus de répondre*

E.3.1. *Si " Autre " pour E.3.:* Veuillez préciser.

- _________
- *Ne sait pas*
- *Refus de répondre*

E.4. Concernant votre rôle de ASC, qui contactez-vous actuellement pour communiquer et signaler les problèmes ? *(Question à choix multiple)*

- Chef CSB/CHRD
- Agents de santé basés dans les centres de santé autres que les chefs CSB/CHRD
- Inspecteur médical
- Chef à domicile

- Travailleurs d'ONG
- Autre
- Je n'ai pas de grande relation
- *Ne sait pas*
- *Refus de répondre*

E.4.1. *Si " Autre " pour E.4. :* Veuillez préciser.

- o _________
- o *Ne sait pas*
- o *Refus de répondre*

F. **Motivation**

Je vais maintenant vous présenter la déclaration d'autres ASC sur leurs motivations. Veuillez indiquer dans quelle mesure vous êtes d'accord ou en désaccord avec ces déclarations. *Pour les questions suivantes : utilisez les émoticônes de la dernière page pour collecter des réponses à choix multiples. Dites aux participants de montrer l'image qui correspond le mieux à ce qu'ils ressentent lorsqu'ils entendent le mot*

F.1. "M'engager **bénévolement** en tant qu'ASC est très important pour moi."

- o Entièrement d'accord
- o D'accord
- o Ni d'accord ni en désaccord
- o En désaccord
- o Entièrement en désaccord
- o *Ne sait pas*
- o *Refus de répondre*

F.2. "M'engager **bénévolement** en tant qu'ASC est très important pour la communauté que je sers."

- o Entièrement d'accord
- o D'accord
- o Ni d'accord ni en désaccord
- o En désaccord
- o Entièrement en désaccord
- o *Ne sait pas*
- o *Refus de répondre*

F.3. "Des **avantages non monétaires** jouent un rôle important dans ma motivation en tant qu'ASC."

- Entièrement d'accord
- D'accord
- Ni d'accord ni en désaccord
- En désaccord
- Entièrement en désaccord
- *Ne sait pas*
- *Refus de répondre*

F.4. "La **reconnaissance** de ma communauté joue un grand rôle dans ma motivation en tant qu'ASC."

- Entièrement d'accord
- D'accord
- Ni d'accord ni en désaccord
- En désaccord
- Entièrement en désaccord
- *Ne sait pas*
- *Refus de répondre*

F.5. "L'argent que je gagne en tant qu'ASC est essentiel pour **couvrir les besoins de mon ménage**."

- Entièrement d'accord
- D'accord
- Ni d'accord ni en désaccord
- En désaccord
- Entièrement en désaccord
- *Ne sait pas*
- *Refus de répondre*

F.6. "Un **contrat de travail longue durée** en tant qu'ASC me motivera encore plus à exercer mon travail de manière régulière."

- Entièrement d'accord
- D'accord
- Ni d'accord ni en désaccord
- En désaccord
- Entièrement en désaccord
- *Ne sait pas*

- *Refus de répondre*

F.7. "Recevoir un **salaire fixe régulier** me motivera davantage à faire mon travail de ASC."

- Entièrement d'accord
- D’accord
- Ni d'accord ni en désaccord
- En désaccord
- Entièrement en désaccord
- *Ne sait pas*
- *Refus de répondre*

F.8. "Un **CDI** (contrat à durée indéterminé) en tant qu'ASC me poussera plus loin, MÊME si cela peut signifier que les revenus de mon ménage seront diminués."

- Entièrement d'accord
- D’accord
- Ni d'accord ni en désaccord
- En désaccord
- Entièrement en désaccord
- *Ne sait pas*
- *Refus de répondre*

F.9. "Je suis prêt à consacrer plus de temps à mon rôle de AC si cela me permet de gagner un **revenu prévisible**."

- Entièrement d'accord
- D’accord
- Ni d'accord ni en désaccord
- En désaccord
- Entièrement en désaccord
- *Ne sait pas*
- *Refus de répondre*

G. **Revenu**

Je vais maintenant vous poser quelques questions concernant les incitatifs potentiels que vous pourriez recevoir en tant qu'ASC.

G.1. Au cours des deux dernières années, en tant qu'ASC, quels incitatifs avez-vous reçus de la part d'un acteur de la santé, tels qu'un CSB, une ONG ou tout autre organisme ?*(Question à choix multiple)*

- Rémunération/Incitatif financier
- Assistance en direct
- Transport (par exemple vélo, frais de transport)
- Soutien alimentaire/nutritionnel
- Expression de gratitude/reconnaissance
- Supervision
- Formation
- Réunion
- Produits de santé
- Autre
- Aucun
- *Ne sait pas*
- *Refus de répondre*

G.1.1. Si " Autre " pour G.1 : Veuillez préciser.

- o _________
- o *Ne sait pas*
- o *Refus de répondre*

G.2. Au cours des deux dernières années, en tant qu'ASC, quel incitatif avez-vous reçu de la part de la communauté ? *(Question à choix multiple)*

- Rémunération / Incitatif financier
- Assistance en direct (famaACna)
- Transport (par exemple vélo)
- Soutien nutritionnel
- Expression de gratitude / reconnaissance
- Autre
- Aucun
- *Ne sait pas*
- *Refus de répondre*

G.2.1. Si " Autre " pour G.2. : Veuillez préciser.

- _________
- *Ne sait pas*
- *Refus de répondre*

G.3. Avez-vous un emploi à temps plein autre que votre emploi en tant qu'ASC ?

- o Oui

- Non
- *Ne sait pas*
- *Refus de répondre*

G.3.1. *Si " Oui " pour G.3.* : Quelle est votre profession ?

- Cadre
- Employé avec qualification
- Employé sans qualification
- Étudiant
- Autre
- *Ne sait pas*
- *Refus de répondre*

G.3.1.1. Si " Autre " pour G.3.1. : Veuillez préciser.

- _________
- *Ne sait pas*
- *Refus de répondre*

H. **À propos du participant**

Enfin, j'ai quelques questions sur votre parcours.

H.1. Pour quel type d'institution avez-vous travaillé au cours des deux dernières années ? *(Question à choix multiple)*

- Établissement de santé publique
- Établissement de santé confessionnel
- ONG
- Autre
- *Ne sait pas*
- *Refus de répondre*

H.1.1. *Si " Autre " pour H.1. :* Veuillez préciser.

- _________
- Ne sait pas
- Refus de répondre

*H.*2. Quel âge avez-vous?

- _________ ans

- *Ne sait pas*
- *Refus de répondre*

H.4. Quel est votre état civil ?

- Célibataire
- En concubinage
- Marié
- Officiellement divorcé
- Séparé
- Veuf
- *Ne sait pas*
- *Refus de répondre*

H.5. Combien de personnes, y compris vous-même, vivent dans votre foyer ?

- ________________
- *Ne sait pas*
- *Refus de répondre*

H.6. Quelle est la classe d'école la plus élevée que vous avez fréquentée ?

- Je ne suis pas allé à l'école
- Niveau de base
- Secondaire
- Supérieur (par exemple université)
- *Ne sait pas*
- *Refus de répondre*

H.6.1. Si " Je ne suis pas allé à l'école " ou " Niveau de base " pour H.6. : Pourriez-vous s'il vous plaît lire les deux phrases suivantes ? (Demandez au participant de lire à haute voix les phrases préparées et de donner une estimation basée sur vos observations)

"TAHIONAO RY ZANAHARY ITY NOSY MAMINAY ITY, HIADANA SY HO FINARITRA!
HE SAMBATRA MARINA IZAHAY"

- Avec grande difficulté
- Avec difficulté
- Avec facilité
- Avec grande facilité
- Refus de lire

H.6.2. Si “ Je ne suis pas allé à l’école “ ou “ Niveau de base “ pour H.6. : Pouvez-vous faire le calcul suivant ?

“XXXXXXXX”

- Oui
- Non
- *Refus de répondre*

H.7. Quel est le revenu mensuel moyen de votre foyer ?

- __________Ar
- *Ne sait pas*
- *Refus de répondre*

H.8. Combien gagnez-vous en moyenne par an en tant qu'ASC

? O < 20.000 Ariary

O 20.000 - 50.000 Ariary

O 50.000 - 100.000 Ariary

O 100.000 - 200.000 Ariary

O 200.000 - 500.000 Ariary

O 500.000 - 1.000.000 Ariary

O Autre

O Ne sait pas

O Refus de répondre

H.8.1. Si autre, précisez :

_ _ _ _ _ _ _ _

- *Ne sait pas*
- *Refus de répondre*

H.9. Dans quelle région travaillez-vous en tant qu'ASC ?

- Analamanga
- Anosy
- Atsimo Andrefana
- Androy
- Autre
- *Ne sait pas*
- *Refus de répondre*

H.9.1. Si " Autre " pour H.9. : Veuillez préciser.

- __________
- Ne sait pas
- Refus de répondre

H.10. Travaillez-vous principalement en tant qu'ASC en zone rurale ou urbaine ?

- Zone rurale
- Zone urbaine
- Les deux
- *Ne sait pas*
- *Refus de répondre*

H.11. Avez-vous un téléphone que vous pouvez utiliser ?

- Oui, un téléphone personnel
- Oui, un téléphone familial, mais pas un téléphone personnel
- Oui, un taxi phone
- Autre
- Non
- *Ne sait pas*
- *Refus de répondre*

H.11.1. Si " Autre " pour H.11. : Veuillez préciser.

- __________
- *Ne sait pas*
- *Refus de répondre*

H.12. Avez-vous déjà entendu parler du Mobile Money ?

- Oui
- Non
- *Ne sait pas*
- *Refus de répondre*

H.13. Avez-vous déjà utilisé un service Mobile Money ?

- Oui
- Non
- *Ne sait pas*
- *Refus de répondre*

C'est la fin de l'entretien. Merci pour vos réponses. Ils ont été très utiles.

Questionnaire en malagasy

Andiam-panontaniana

Andiamam-panontaniana momba Mpanentana ara-pahasalamana eto Madagasikara

A. **Fanekena an-tsitrapo sy maso-tsivana**

A.1. Nampahafantarina ve ianao fa an-tsitrapo ny fandraisana anjara amin'ny asa fanadihadiana?

- ○ Resy lahatra amin'ny asa fanadihadiana ilay olona ary nanaiky handray anjara
- ○ Tsy manaiky handray anjara ilay olona

A. Lahy sa Vavy ny mpandray anjara (jerena fa tsy hanontaniana)

A.2. 18 taona no mihoatra ve ianao?

- ○ Eny
- ○ Tsia

A.3. Mpanentana ara-pahasalamana niasa erin-taona mihoatra ve ianao?

- ○ Eny
- ○ Tsia

B. **Tontolon'ny asa atao**

B.1. Toy ny ahoana no nahalasa anao ho mpanentana ara-pahasalamana? *(afaka misy valiny maro)*

- ● Natolotra mpanentana ara-pahasalamana
- ● Nosafidin'ny Sefo Fokontany
- ● Nolanian'ny mponina (fivoriana)
- ● Nosafidin'ny Ben'ny tanana na komity misahana ny ara-sosialy (Kaominina)
- ● Nosafidin'ny ONG
- ● Nosafidin'ny raiman-dreny ara-pinoana
- ● Nosafidin'ny mpiasan'ny fahasalamana
- ● *hafa*
- ● *Tsy mahafantatra*
- ● *Tsy te hamaly*

B.1.1. Raha hafa ny B.1. : Marihina.

- ○ ___________
- ○ *Tsy mahafantatra*
- ○ *Tsy te hamaly*

B.2. Amin'ny maha mpanentana ara-pahasalamana anao, misahana asa sosialy hafa ve ianao?

- Eny
- Tsia
- *Tsy mahafantatra*
- *Tsy te hamaly*

B.2.1. Raha Eny, amin'ny sehatra inona ?

- _ _ _ _ _ _ _ _ _ _ _ _
- *Tsy mahafantatra*
- *Tsy te hamaly*

B.2' Nisy fampafantarana anao amin'ny fomba ofisialy ho anisan'ny mpanentana ara-pahasalamana eo anivon'ny fiarahamonina misy anao ve?

O Eny

O Tsia

O Tsy mahafantatra

O Tsy te hamaly

B.3. Firy taona izay naha mpanentana ara-pahasalamana anao?

- __________ taona
- *Tsy mahafantatra*
- *Tsy te hamaly*

B.4. Firy ny isan'ny Fokontany iandreketanao?

- _____________
- *Tsy mahafantatra*
- *Tsy te hamaly*

B.5. Araka ny maha mpanentana ara-pahasalamana anao, toy ny ahoana ny fotoana isanao mahazatra?

- Isan'andro
- Andro manokana ao anatin'ny herinandro
- Miovaova araka ny fahafahana omen'ny asa(ko) manokana
- Miovaova araka ny fahafahana apetraky antony ankoatra ny asa
- *Tsy mahafantatra*
- *Tsy te hamaly*

B.5.1. Raha miovaova araka ny fahafahana apetraky antony ankoatra ny asa, marihina.

- __________
- *Tsy mahafantatra*

- *Tsy te hamaly*

B.6. Adiny firy isan-kerinandro no atokanao amin'ny asa ataon'ny mpanentana ara-pahasalamana ? *(isa feno)*

- __________ ora
- *Tsy mahafantatra*
- *Tsy te hamaly*

B.7. Toy ny ahoana ny hiazohanao ny olona hiandreketanao?Comment accédez-vous aux communautés dont vous êtes responsable ? *(Afaka misy valiant maromaro)*

- tongotra
- bisikileta
- fiara iombonana
- lakana
- fitaterana hafa
- *Tsy mahafantatra*
- *Tsy te hamaly*

B.7.1. Raha hafa, marihina.

- _________
- *Tsy mahafantatra*
- *Tsy te hamaly*

B.8. Inona avy ny fitaovana fampiasanao amin'ny fanatanterahana ny asa maha mpanentana ara pahasalamana anao? *(Afaka misy valiny maromaro)*

- Fitaovana fanentanana/fampianarana (sary, boky)
- Toro-lalana ho an'ny asa, boky mirakitra ny atao rehetra
- Trosy vonjy taitra
- Kitapo fibaby
- Boky mirakitra ny fanaraha-maso atao
- Akanjo voatokana (zile, kaskety)
- Bisikileta
- Finday
- Fanamafisa-peo ("Mégaphone")
- Hafa
- Tsy misy fitaoavana
- *Tsy mahafantatra*
- *Tsy te hamaly*

B.8.1. Raha hafa, marihina.

- _________
- *Tsy mahafantatra*

○ *Tsy te hamaly*

B.9. Inona avy ireo paik'ady entinao manatanteraka ny asa tao anatin'ny roa taona farany? *(Afaka misy valiny maro)*

● fihaonana amin'ny tsirairay (fitsidihana isan-tokatrano)
● fitafatafana amin'ny vondron'olona (fivoriana)
● " Counseling "
● fanentanana faobe (mégaphone, " Tam Tam ")
● Hafa
● *Tsy mahafantatra*
● *Tsy te hamaly*

B.9.1. Raha hafa, marihina.

○ _________
○ *Tsy mahafantatra*
○ *Tsy te hamaly*

C. **Asa atao**

Amin'izao fizarana izao dia hiresaka mikasika ny asa niandreketanao nandritra ny roa taona farany isika:

C.1. Amin'ny maha mpanentana ara-pahasalamana anao, nandray anjara tamin'ny fisorohana na fiadiana ny raboka ve ianao tato anatin'ny roa taona?

○ Eny
○ Tsia
○ *Tsy mahafantatra*
○ *Tsy te hamaly*

C.1.1. Raha Eny ny C.1.: Inona amin'ireo asa mifandraika amin'ny tiberkilaozy no efa nataonao tato anatin'ny roa taona? *(Afaka misy valiant maromaro)*

● Manentana ny fisorohana ny tiberkilaozy
● Manentana mikasika ny fifindran'ny tiberkilaozy
● Manentana mikasika ny fisehon'ny tiberkilaozy
● Manentana ny mponina mikasika ny maha maimaipoana ny fitilina sy ny fanafodin'ny tiberkilaozy
● Mijery izay ahiahiana no voan'ny tiberkilaozy ary mandefa azy ho raisin-tanana ● Mijery izay nifanerasera tamin'ny ahiahiana ho voan'ny tiberkilaozy ary mandefa azy ho raisin-tanana
● Miady amin'ny fanavakavahana sy fanilihana ny voan'ny tiberkilaozy
● Manentana ny amin'ny hanaovana vaksiny BCG ny zaza latsaky ny 45 andro
● Miandraikitra ny fanaraha-maso ny fihinanan'ny marary fanafody
● Misolo-tena ireo rafitra mitily sy mitsabo ny tiberkilaozy eo amin'ny fiarahamonina
● Mampita vaovao amin'ny rafitra mitily sy mitsabo ny tiberkilaozy
● Mitady sy manara-maso ireo tsy mpiala an-daharana

● Manentana ireo marary hanao fitilina VIH/SIDA
● Hafa
● *Tsy mahafantatra*
● *Tsy te hamaly*

C.1.1.1. Raha hafa ny C.1.1., marihina.

○ _________
○ *Tsy mahafantatra*
○ *Tsy te hamaly*

C.2. Nandray anjara amin'ny fisorohana na fandraisana an-tanana VIH/SIDA ve ianao tato anatin'ny roa taona?

○ Eny
○ Tsia
○ *Tsy mahafantatra*
○ *Tsy te hamaly*

C.2.1. Raha Eny ny C.2.: Inona amin'ireo asa manodidina ny VIH/SIDA no nataonao tao anatin'ny roa taona farany? *(Afaka misy valiny maro)*

● Manentana amin'ny fisorohana ny VIH/SIDA
● Manentana amin'ny fifindran'ny VIH/SIDA
● Manentana amin'ny trangan'aretina misy amin'ny VIH/SIDA
● Manentana ny fiarahamonina amin'ny maha-maimaipoana ny fitilina sy fitsabona ny VIH/SIDA
● Manentana ny mponina hanao fitilina raha misy tranga hafahafa amin'ny taovam-pananahana
● Mitily sy mandefa ireo hatahorana voan'ny VIH/SIDA handraisana an-tanana azy ireo
● Mitily sy mandefa ireo niaraka tamin'ny olona voan'ny VIH/SIDA handraisana an-tanana azy ireo
● Miady amin'ny fanavakavahana sy fanilihana an'ireo olona mitondra ny tsimok'aretina VIH/SIDA
● Misolo tena ireo mpiasan'ny fahasalamana mpitily sy mpandray an-tanana io aretina io eo amin'ny fiarahamonina
● Mampita ny vaovao misy amin'ireo mpiasan'ny fahasalamana mpitily sy mpandray an-tanana
● Hafa
● *Tsy mahafantatra*
● *Tsy te hamaly*

C.2.1.1. Raha hafa ny C.1.2.1.: marihina.

○ _________
○ *Tsy mahafantatra*
○ *Tsy te hamaly*

C.3. Ao anatin'ny asa maha mpanentana ara-pahasalamana, nandray anjara tamin'ny fisorohana sy fifehezana ny tazomoka ve ianao tato anatin'ny roa taona farany?

- Eny
- Tsia
- *Tsy mahafantatra*
- *Tsy te hamaly*

C.3.1. Raha Eny ny C.3.: Inona avy ireo asa mifandraika amin'ny tazomoka natonao tato anatin'ny roa taona? *(afaka misy valiny maro)*

- Manentana ny vehivavy mitondra vohoka hihinana ny fanafody fisorohana farafahakeliny in telo fatra telo (TPI)
- Mamamntatra ny trangan'aretin'ny tazomoka
- Manentana amin'ny ady amin'ny moka
- Mizara lay misy ody moka ary mampahafanatatra ny fiarahamonina mikasika ny maha zava dehibe hilana izany
- Fanaovana ny test TDR ary manome ny fitsaboana ho an'ny tazomoka tsy misy fahasarotany, manome ny ACT
- Mandefa ny olona tratry ny tazomoka misy fahasarotany any amin'ny tobim-pahasalamana
- Hafa
- *Tsy mahafantatra*
- *Tsy te hamaly*

C.3.1.1. Raha hafa ny C.3.1., marihina.

- _________
- *Tsy mahafantatra*
- *Tsy te hamaly*

C.4. Amin'ny asa maha mpanentana ara-pahasalamana, misy areti-mifindra hafa niasanao ve tato anatin'ny roa taona farany? *(Afaka misy valiant maro)*

- Aretina azo avy amin'ny firaisana ara-nofo
- COVID-19
- Haromotana
- Cysticercose, filariôzy, bilarziôzy, kankana
- Pesta
- Fandraisana anjara amin'ny fanomezana fanafody faobe (filariôzy, bilarziôzy)
- Tsy miasa amin'ny areti-mifindra hafa
- Hafa
- *Tsy mahafantatra*
- *Tsy te hamaly*

C.4.1. Raha hafa ny C.4., marihina.

- _________
- *Tsy mahafantatra*
- *Tsy te hamaly*

C.5. Ny fanarahamaso ifotony dia endrika iray ny asa atao hijerena ary ahafahana mampahafantatra

izay tranga ara-pahasalamana misy eo amin'ny fiarahamonina.

Ao anatin'ny asa maha mpanentana ara-pahasalamana anao, nandray anjara tamin'ny fanarahamaso ifotiny ve ianao tato anatin'ny roa taona?

- o Eny
- o Tsia
- o *Tsy mahafantatra*
- o *Tsy te hamaly*

C.5.1. Raha Eny ny C.5.: Inona amin'ireo asa fanarahamaso ifotiny no nataonao tato anatin'ny roa taona farany? *(Afaka misy valiny maro)*

● Mamantatra ireo aretina, tranga ara-pahasalamana misy eo amin'ny fiarahamonina izay tatao ratsy ho an'ny fahasalamam-bahoaka ary manara-maso izay ao anatin'ny firaketana an-tsoratra ● Mandray anjara amin'ny fanadihadiana ny antom-pahafatesana
● Mitatitra ny fahafatesana
● Izay antom-pahafatesana na aretina tsy ampoizina dia taterina amin'ny tobim-pahasalamana fototra
● Mampandray anjara ny mpitondra ifotony amin'ny fanazavana ny tranga ara-pahasalamana sy fiparitahan'aretina eo anivon'ny fiarahamonina
● Fanohanana ny mpiasan'ny fahasalamana mandritra ny fanadihadiana raha misy fiparitahan'aretina sy fitadiavana ny olona izay nikasoka tamin'izany
● Mandray anjara amin'ny fanaovana sari-tany ny asa sy tatao ara-pahasalamana
● Hafa
● *Tsy mahafantatra*
● *Tsy te hamaly*

C.5.1.1. Raha hafa, marihina.

- o _________
- o *Tsy mahafantatra*
- o *Tsy te hamaly*

C.6. Ao anatin'ny asa maha mpanentana ara-pahasalamana , nandray anjara tao anatin'ireto ve ianao tato anatin'ny roa taona farany?

● Fahasalaman'ny adolantsento sy tanora (10-20 taona)
● Fandrindram-piterahana
● Fanarahamaso mandritra ny vohoka (fotoana mialoha ny fiterahana)
● Fanarahamaso ny reny sy ny zaza vao teraka (aorian'ny fiterahana sy 0-42 andro amin'ny zaza vao teraka)
● Vaksiny
● Fanjarina ara-tsakafo (fanarahamaso ny fitomboana, fizarana vitamine A, …)
● Firaketana ny fiterahana
● Fivoarana mandritra ny fahazazana
● Fanarahamaso ny zaza marary
● Fampahafantarana sy fisorohana ny aretina mpahazo ny zaza

- “ WASH ” (rana, fidiovana sy fanadiovana)
- Fandrindrana, fisian’ny fitaovana ampiasaina amin’ny asan’ny fahasalamana
- Fikirakirana ireo tsara ho fantatra ho an’ny fanarahamaso sy evaluation
- Fanarahamaso ny fahasalamana (loza, fiparitahan’aretina, fanapozinana)
- Vonjy taitra
- Fanomezan-dra
- Tsy misy amin’ireo voalaza ireo
- *Tsy mahafantatra*
- *Tsy te hamaly*

C.7. Amin’ny asa fanentanana ara-pahasalamana, aretina tsy mifindra inona no niasanao tato anatin’ny roa taona farany? *(Afaka misy valiny maro)*

- Fiakaran’ny tosi-dra
- Diabeta
- Homamiadan’ny vozon’ny tranon-jaza na ny nono
- Fahavoazan’ny taovam-pisefoana maharitra
- Hafa
- *Tsy mahafantatra*
- *Tsy te hamaly*

C.7.1. Raha hafa ny C.7., marihina.

- ________
- *Tsy mahafantatra*
- *Tsy te hamaly*

C.8. Mbola misy asa ataonao amin’ny asa fanentanana ara-pahasalamana mbola tsy noresahan-tsika ve?

- Eny
- Tsia
- *Tsy mahafantatra*
- *Tsy te hamaly*

C.8.1. Raha Eny ny C.8., Marihina.

- ________
- *Tsy mahafantatra*
- *Tsy te hamaly*

D. **Fihofanana**

D.1. Tato anatin’ny roa taona farany, nisitraka fihofanana iray andro farafaharatsiny na mihoatra ve ianao?

- Eny
- Tsia
- *Tsy mahafantatra*

- *Tsy te hamaly*

D.1.1. Raha Eny ny D.1. : Inona avy no noresahina tamin'ny fiofanana nandraisanao anjara tato anatin'ny roa taona farany? *(Afaka misy valiny maro)*

- Ireo andraikitry ny Mpanentana ara-pahasalamana
- Manentana amin'ireo fitondran-tena prioritera
- Fandrindram-piterahana
- Fanarahamaso ny reny sy ny zaza
- Vaksiny
- Ady amin'ny fanaovana dokotera tena
- Fifehezana ny aretina miparitaka
- Fisorohana ny areti-mifinra
- Aretina hafa
- Lozam-pifamoivozana
- Fanentanana " WASH "
- Fandrindrana fanateram-panafody
- Fandrindrana ny fandraisana an-tanana ifotiny
- Hafa
- *Tsy mahafantatra*
- *Tsy te hamaly*

D.1.1.1. Raha hafa ny D.1.1., marihina.

- __________
- *Tsy mahafantatra*
- *Tsy te hamaly*

D.1.2. Iza no nikarakara sy namatsy vola ny fandraisanao anjara tamin'izany fiofanana izany? *(Valiny maro)*

- Tobim-pahasalamana
- ONG
- Hôpitaly lehibe eo anivon'ny distrika
- Ministeran'ny fahasalamam-bahoaka
- PNLT
- Hafa
- *Tsy mahafantatra*
- *Tsy te hamaly*

D.1.2.1. *Raha hafa ny D.1.2.:* marihina.

- __________
- *Tsy mahafantatra*
- *Tsy te hamaly*

E. **Fanarahamaso sy fiantsorohana**

E.1. Manana lehibe mpanara-maso ve ianao ?

- *Eny*
- *Tsia*
- *Tsy mahafantatra*
- *Tsy te hamaly*

E.1.1. *Raha Eny ny E.1. : Iza no mpanara-maso napetraky ny rafitra ny asa maha mpanentana ara pahasalamana anao? (Afaka misy valiny maro)*

- Lehiben'ny tobim-pahasalamana CSB/CHRD
- Mpiasan'ny fahasalamana eo anivon'ny CSB na CHRD ankoatra ireo lehibe ao
- Médecin inspecteur
- Chef Fokontany
- Mpanara-maso avy amin'ny ONG
- Hafa
- Tsy manana mpanaraka ny asa vita aho
- *Tsy mahafantatra*
- *Tsy te hamaly*

E.1.1.1. *Raha hafa ny E.1.1.:* marihina

- _________
- *Tsy mahafantatra*
- *Tsy te hamaly*

E.2. Iza no tena manaramaso ny asa ataonao amin'ny maha mpanentana ara-pahasalamana anao?

- Sefo CSB/CHRD
- Mpiasan'ny fahasalamana eo anivon'ny CSB na CHRD ankoatra ireo lehibe ao
- Médecin Inspecteur
- Mpiasan'ny ONG
- Hafa
- Tsy manana olona tena mpanaramaso aho
- *Tsy mahafantatra*
- *Tsy te hamaly*

E.2.1. *Raha hafa ny E.2.,* marihina.

- _________
- *Tsy mahafantatra*
- *Tsy te hamaly*

E.3. Araka ny hevitrao, inona avy ireo tokony hataon'ny lehibenao mba hanohanana anao bebe kokoa amin'ny asa maha mpanentanamapnentana ara-pahasalamana anao?quelles tâches devrait assumer votre superviseur pour mieux vous soutenir dans votre rôle d'ASC ? *(Afaka valiny maromaro)*

- Fandrindrana ny asa
- Fanomezana fitaovana anaovana ny asa

- Manome toromarika fanatanterahana ny asa
- Manoro hevitra
- Fitsarana ny asa vita
- Mandamina ny disadisa
- Mampifandray amin'ny departemanta hafa misy
- Fanarahamaso
- Fanomezana fanohanana (incitatif)
- Hafa
- *Tsy mahafantatra*
- *Tsy te hamaly*

E.3.1. *Raha ny E.3.:* marihina.

- ○ _________
- ○ *Tsy mahafantatra*
- ○ *Tsy te hamaly*

E.4. Mikasika ny asan'ny mpanentana ara-pahasalamana, iza ny antsoina hampitana ny olana?

(Afaka misy valiny maro)

- Sefo CSB/CHRD
- Mpiasan'ny fahasalamana eo anivon'ny CSB na CHRD ankoatra ireo lehibe ao
- Médecin Inspecteur
- Mpiasan'ny ONG
- Hafa
- Tsy manana olona tena mpanaramaso aho
- *Tsy mahafantatra*
- *Tsy te hamaly*

E.4.1. *Raha hafa ny E.4.:* marihina.

- ○ _________
- ○ *Tsy mahafantatra*
- ○ *Tsy te hamaly*

F. **Fahazotoana sy tambin-karama**

Hilaza aminao ny fanambaran'ny mpanentana ara-pahasalamana hafa momba ny antony manosika azy ireo aho izao. Lazao azafady hoe toy ny ahoana ny fanekenao na tsy fanekenao ireo fanambarana ireo.

F.1. "**manan-danja amiko manokana ny fanatanterahana ny andraikitro amin'ny maha mpirotsaka an-tsitrapo ny mpanentana ara-pahasalamana**"

- ○ Miombon-kevitra tanteraka
- ○ Miombon-kevitra
- ○ Sady tsy manaiky no tsy mandà

- tsy miombon-kevitra
- Tena tsy miombon-kevitra
- *Tsy mahafantatra*
- *Tsy te hamaly*

F.2. **"Zava-dehibe ho an'ny vondrom-piarahamonina sahaniko ny fanatanterahana ny andraikitro maha mpanentana ara-pahasalamana mpirotsaka an-tsitrapo ahy."**

- Miombon-kevitra tanteraka
- Miombon-kevitra
- Sady tsy manaiky no tsy mandà
- tsy miombon-kevitra
- Tena tsy miombon-kevitra
- *Tsy mahafantatra*
- *Tsy te hamaly*

F.3. **"Ny tombontsoa tsy ara-bola dia manana anjara toerana lehibe amin'ny fazotoako amin'ny maha mpanentana ara-pahasalamana ahy."**

- Miombon-kevitra tanteraka
- Miombon-kevitra
- Sady tsy manaiky no tsy mandà
- tsy miombon-kevitra
- Tena tsy miombon-kevitra
- *Tsy mahafantatra*
- *Tsy te hamaly*

F.4. **"Ny fankasitrahana avy amin'ny fiarahamonina dia mitana anjara toerana lehibe amin'ny fahazotoako amin'ny maha mpanentana ara-pahasalamana ahy.""**La **reconnaissance** de ma communauté joue un grand rôle dans ma motivation en tant qu'ASC."

- Miombon-kevitra tanteraka
- Miombon-kevitra
- Sady tsy manaiky no tsy mandà
- tsy miombon-kevitra
- Tena tsy miombon-kevitra
- *Tsy mahafantatra*
- *Tsy te hamaly*

F.5. **"Ny vola azoko amin'ny maha mpanentana ara-pahasalamana ahy dia tena ilaina amin'ny fiainako andavanandro ao an-tokatranoko."**

- Miombon-kevitra tanteraka
- Miombon-kevitra

- Sady tsy manaiky no tsy mandà
- tsy miombon-kevitra
- Tena tsy miombon-kevitra
- *Tsy mahafantatra*
- *Tsy te hamaly*

F.6. **Ny fifanarahana arak'asa maharitra amin'ny maha mpanentana ara-pahasalamana dia hanosika ahy bebe kokoa amin'ny fanatanterahana ny asako."**

- Miombon-kevitra tanteraka
- Miombon-kevitra
- Sady tsy manaiky no tsy mandà
- tsy miombon-kevitra
- Tena tsy miombon-kevitra
- *Tsy mahafantatra*
- *Tsy te hamaly*

F.7. **"Ny fandraisana karama raikitra tsy tapaka dia hanosika ahy bebe kokoa amin'ny fanatanterahana ny asako amin'ny maha mpanentana ara-pahasalamana ahy."**

- Miombon-kevitra tanteraka
- Miombon-kevitra
- Sady tsy manaiky no tsy mandà
- tsy miombon-kevitra
- Tena tsy miombon-kevitra
- *Tsy mahafantatra*
- *Tsy te hamaly*

F.8. **"Ny fifanarahana arak'asa tsy voafetra amin'ny maha mpanentana ara-pahasalamana dia hanosika ahy bebe kokoa, NA dia mety hidika aza izany fa ho kely kokoa ny fidiram-bolako."**

- Miombon-kevitra tanteraka
- Miombon-kevitra
- Sady tsy manaiky no tsy mandà
- tsy miombon-kevitra
- Tena tsy miombon-kevitra
- *Tsy mahafantatra*
- *Tsy te hamaly*

F.9. **"Vonona aho hanokana fotoana bebe kokoa amin'ny andraikitro amin'ny maha mpanentana ara-pahasalamana ahy raha toa ka mahazo fidiram-bola azo antoka aho amin'ny alalan'izany."**

- Miombon-kevitra tanteraka
- Miombon-kevitra
- Sady tsy manaiky no tsy mandà
- tsy miombon-kevitra

- Tena tsy miombon-kevitra
- *Tsy mahafantatra*
- *Tsy te hamaly*

G. **Fidiram-bola**

G.1. Tao anatin'ny 2 taona farany, inona amin'ireto no efa azonao avy amin'ny tobim-pahasalamana na mpisehatra amin'ny fahasalamana (oh: CSB, ONG, na hafa)?

(Afaka misy valiny maro)

- Tambin-karama
- Fanohanana mivantana (famaACna)
- Fitaterana (oh: bisikileta, saran-dalana)
- Fanohanana ara-tsakafo/sakafo
- Maneho fankasitrahana
- Fanaraha-maso nohatsaraina
- Fiofanana
- FIVORIANA
- Fanafody sy fitaovam-pitsaboana
- Hafa
- *Tsy mahafantatra*
- *Tsy te hamaly*

G.1.1. Raha hafa, marihina

- __________
- *Tsy mahafantatra*
- *Tsy te hamaly*

G.2. Tato anatin'ny 2 taona farany, efa nahazo "motivation" amin'ny maha mpanentana ara pahasalamana ve ianao avy amin'ny fiaraha-monina?

(Afaka misy valiny maro)

- Tambin-karama
- Fanohanana mivantana
- Fitaterana (oh: bisikileta)
- Fanohanana ara-tsakafo
- Fankasitrahana
- Hafa
- *Tsy mahafantatra*
- *Tsy te hamaly*

Raha hafa dia marihina,

- __________
- *Tsy mahafantatra*
- *Tsy te hamaly*

G.3. Manana asa ve ianao ankoatra ny asa ataonao amin'ny maha mpanentana ara-pahasalamana anao?

- o Eny
- o Tsia
- o *Tsy mahafantatra*
- o *Tsy te hamaly*

G.3.1. Raha Eny : Inona no sokajy misy ny asanao ?

- o Mpiasa ambony
- o Mpiasa vita fihofanana
- o Mpiasa vita tsy fihofanana
- o Mpianatra
- o Hafa
- o *Tsy mahafantatra*
- o *Tsy te hamaly*

H. Mombamomba ny mpandray anjara

Farany, manana fanontaniana vitsivitsy momba anao aho.

E.1. Inona no niasanao amin’ny maha mpanentana ara-pahasalamana anao nandritra ny 2 taona farany? Azafady tsidiho izay rehetra mihatra.

(Afaka misy valiny maro)

- Tobim-pahasalamana
- Toeram-pitsaboana mifototra amin'ny finoana
- ONG
- Hafa
- *Tsy mahafantatra*
- *Tsy te hamaly*

E.1.1. Raha hafa, marihina:

_ _ _ _ _ _ _ _

- o *Tsy mahafantatra*
- o *Tsy te hamaly*

E.2. Firy taona ianao?

- o _________ taona
- o *Tsy mahafantatra*
- o *Tsy te hamaly*

E.4. Sata ara-panambadiana?

- o Tokatena

- Tokatrano imaso
- manambady
- nisara-panambadiana ara-panjakana
- nisara-bady
- maty vady
- *Tsy mahafantatra*
- *Tsy te hamaly*

E.5. Firy ianareo no miara-mipetraka ao anatin'ny tokan-trano ?

- ________________
- *Tsy mahafantatra*
- *Tsy te hamaly*

E.6. Hatraiza ny fianarana vitanao?

- Tsy nianatra
- Ambaratonga fototra
- Ambaratonga faharoa
- Ambaratonga ambony
- *Tsy mahafantatra*
- *Tsy te hamaly*

E.6.1. Raha tsy nianatra na hatreo amin'ny ambaratonga fototra dia hasaina mamky ny fehezanteny: Afaka mamaky ity fehezanteny ity ve ianao azafady :

"TAHIONAO RY ZANAHARY ITY NOSY MAMINAY ITY, HIADANA SY HO FINARITRA! HE SAMBATRA MARINA IZAHAY"

E.7. Angataho ny mpandray anjara mba hamaky mafy ny fehezanteny ao amin'ny pejy XX ary hanome tombana mifototra amin'ny fandinihanao

- Sahirana be mamaky
- Somary sahirana
- mora
- tena mora
- Tsy mety mamaky

E.8. Mahavita manao kajy ve ianao ?

- Eny
- Tsia
- *Tsy mahafantatra*
- *Tsy te hamaly*

E.9. Ohatrinona ny vola miditra isam-bolana ao an-tokantranonao?

- __________
- *Tsy mahafantatra*
- *Tsy te hamaly*

E.10. Ohatrinona ny vola miditra isam-bolanao amin'ny maha mpanentana ara-pahasalamana anao?

- < 20.000 Ariary
- 20.000 - 50.000 Ariary
- 50.000 - 100.000 Ariary
- Hafa
- *Tsy mahafantatra*
- *Tsy te hamaly*

E.10.1. Raha hafa, marihina

- __________
- *Tsy mahafantatra*
- *Tsy te hamaly*

E.11. Mpanentana ara-pahasalamana amin'ny faritra aiza ianao?

- Analamanga
- Anosy
- Atsimo andrefana
- Androy
- Hafa
- *Tsy mahafantatra*
- *Tsy te hamaly*

E.11.1. Raha hafa, marihina

- __________
- *Tsy mahafantatra*
- *Tsy te hamaly*

E.12. Miasa any ambanivohitra ve ianao sa any an-tanàn-dehibe?

- Ambanivohitra
- An-tanan-dehibe
- Izy roa
- *Tsy mahafantatra*
- *Tsy te hamaly*

E.13. Manana finday azonao ampiasaina ve ianao?

- Eny

- Tsia
- *Tsy mahafantatra*
- *Tsy te hamaly*

E.13.1. Raha Eny, an'iza

- finday ahy manokana
- finday an'ny fianakaviana fa tsy finday manokana
- taxi-phone
- hafa
- *Tsy mahafantatra*
- *Tsy te hamaly*

E.13.2. Raha hafa, marihina

- __________
- *Tsy mahafantatra*
- *Tsy te hamaly*

E.14. Efa naheno momba ny Mobile Money ve ianao?Avez-vous déjà entendu parler du Mobile Money ?

- Eny
- Tsia
- *Tsy mahafantatra*
- *Tsy te hamaly*

E.14.1. Raha Eny, Efa nampiasa tolotra Mobile Money ve ianao?

- Eny
- Tsia
- *Tsy mahafantatra*
- *Tsy te hamaly*

Eto no mifarana ny fanadihadiana. Misaotra anao amin'ny valinteninao. Tena nanampy tokoa izy ireo.
